# LncRNA-Mediated Regulation of Hepatic Metabolic Homeostasis: Cholesterol, Triglyceride, Glucose and Pathological Implications

**DOI:** 10.3390/biology15212029

**Published:** 2026-11-07

**Authors:** Yiteng Zhang, Tongzhan Liu, Yu Li, Guowei Wang, Yiqian Hu, Shichuang Gao, Yu Deng, Derong Xu

**Affiliations:** 1 The QUEEN MARY School, Jiangxi Medical College, Nanchang University, 999 Xuefu Road, Nanchang 330031, China; 4217122147@email.ncu.edu.cn (Y.Z.); 4217122148@email.ncu.edu.cn (T.L.); 2 The National Engineering Research Center for Bioengineering Drugs and Technologies, Jiangxi Province Key Laboratory of Bioengineering Drugs, Institute of Translational Medicine, Jiangxi Medical College, Nanchang University, Nanchang 330103, China; 19921870092@163.com (Y.L.); 15949609587@163.com (G.W.); 419100250032@email.ncu.edu.cn (Y.H.); 18954768290@163.com (S.G.); 407400240002@email.ncu.edu.cn (Y.D.); 3 Jiangxi Academy of Medical Sciences, Nanchang 330006, China

**Keywords:** lncRNA, hepatic metabolism, cholesterol, triglyceride, glucose homeostasis, therapeutic potential

## Abstract

The liver coordinately maintains cholesterol, triglyceride, and glucose homeostasis by integrating the synthesis, storage, utilization, and inter-conversion of these metabolic programs, and their dysregulation contributes to metabolic-associated steatotic liver disease (MASLD), hepatocellular carcinoma (HCC), type 2 diabetes mellitus (T2DM), and gestational diabetes mellitus (GDM). Long non-coding RNAs (lncRNAs), which are not translated into proteins, regulate gene expression through interactions with DNA, RNA, and proteins at transcriptional, post-transcriptional, and epigenetic levels, and have emerged as central determinants of hepatic metabolic balance. This review systematically summarizes how lncRNAs govern cholesterol biosynthesis and efflux, triglyceride synthesis and lipolysis, glycogen turnover, gluconeogenesis, and glycolysis in hepatocytes. Unlike pathway-by-pathway descriptions, we highlight that these programs are coupled through shared signaling networks, including PI3K/AKT/mechanistic target of rapamycin complex 1 (mTORC1), 5′-AMP-activated protein kinase (AMPK), SREBP, PPAR, and FoxO1, and that pleiotropic lncRNAs coordinate lipid and glucose outputs rather than controlling a single isolated process. We further examine how lncRNA dysregulation drives MASLD, HCC, T2DM, and GDM, and evaluate their potential as diagnostic biomarkers and therapeutic targets. Finally, we outline current limitations, including incomplete molecular characterization, low cross-species conservation, heterogeneous experimental validation, and unresolved delivery challenges, which must be addressed before lncRNA-based strategies reach clinical application.

## Simple Summary

Liver diseases related to abnormal fat and sugar metabolism have become one of the most widespread liver health problems worldwide and greatly threaten public health. In this review, we focus on a type of genetic molecule called long non-coding ribonucleic acid, which does not make proteins but controls many biological activities inside liver cells. We aim to summarize how these molecules change fat and sugar processing in the liver and how they contribute to fatty liver disease, liver cancer, and diabetes mellitus. We collected and organized findings from many previous studies and found that these molecules can alter the storage and breakdown of fat and sugar in liver cells, affecting the start and worsening of liver illnesses. Our work shows that these molecules may help identify liver diseases at an early stage or provide new treatment ideas. This knowledge may support future research to develop easier tests and better therapies for patients suffering from metabolic liver diseases.

## 1. Introduction

As a metabolic center of the human body, the liver is indispensable for sustaining the dynamic balance of lipid and glucose metabolism, processes that are tightly orchestrated to ensure the timely supply and utilization of energy substrates for systemic physiological functions [1,2]. In addition to its well-recognized roles in regulating lipids and glucose synthesis, storage, and catabolism, the liver also maintains cholesterol homeostasis, which is critical for membrane biogenesis and steroid hormone synthesis [3]. The dysregulation of these interconnected metabolic pathways not only disrupts hepatic function but also initiates a pathological cascade, and participates in the onset and advancement of metabolic dysfunction-associated steatotic liver disease (MASLD) and hepatocellular carcinoma-conditions that collectively impose a substantial burden on global public health. [4]. Lipids encompass a diverse array of hydrophobic molecules, including cholesterol, fatty acids, triacylglycerols, and phospholipids [5]. These compounds fulfill indispensable roles in numerous biological processes, such as membrane lipid biosynthesis, energy storage and metabolism, and signal transduction [6]. Furthermore, the liver serves as a major site for glucose utilization, facilitating processes like glycogenesis and glycolysis during the post-prandial state, as well as glycogenolysis and gluconeogenesis when blood glucose levels are low [7]. Recently, accumulating evidence has uncovered that lncRNAs act as crucial epigenetic and transcriptional regulators in the hepatic lipid and glucose metabolism [8].

lncRNAs are transcripts longer than 200 nucleotides with no protein-coding capacity. Once dismissed as transcriptional by-products, these molecules are now recognized as critical regulators of a wide range of cellular processes—including chromatin remodeling, transcriptional modulation, and nucleocytoplasmic trafficking [9,10]. Classification schemes for lncRNAs vary widely. By genomic locations, they fall into categories such as intronic, intergenic, sense, antisense, bidirectional, promoter and enhancer-associated lncRNAs (eRNA), whereas based on their regulatory mechanisms, lncRNAs are categorized as decoys, scaffolds, signals, guides, and other subtypes. At the transcriptional and post-transcriptional levels, they modulate gene expression by interacting with DNAs, RNAs, and proteins partners [11]. Notably, emerging evidence indicates that certain lncRNAs contain cryptic open reading frames that encode biologically active micro-peptides, underscoring the breadth of their functional versatility [12,13].

**Figure 1:**
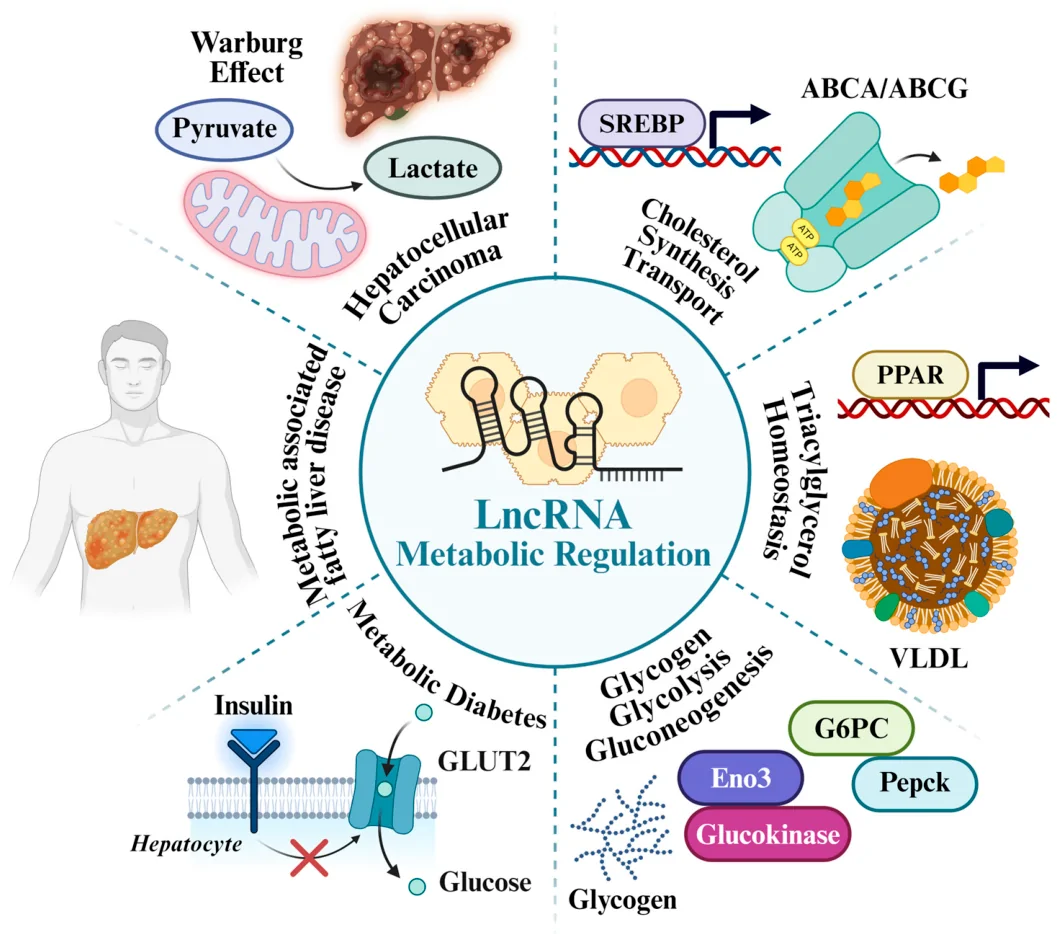
Schematic overview of lncRNA-centered metabolic regulation in the liver. This diagram summarizes the major metabolic pathways modulated by long non-coding RNAs (lncRNAs) in hepatocytes. lncRNAs control insulin-dependent glucose uptake, glycogen turnover, glycolysis and gluconeogenesis, cholesterol and triacylglycerol homeostasis via transcription factors SREBPs and PPARs, as well as the Warburg effect in hepatocellular carcinoma. The dysregulation of lncRNA-dependent metabolic pathways contributes to the pathogenesis of metabolic dysfunction-associated steatotic liver disease and metabolic diabetes. Black solid arrows represent induction or progression, while black blunt-ended inhibitory bars represent inhibitory effects. Cross symbols denote the prevention or impediment of target processes. ABCA/ABCG, ATP-binding cassette transporters; Eno3, enolase 3; G6PC, glucose-6-phosphatase catalytic subunit; GLUT2, glucose transporter 2; PEPCK, phosphoenolpyruvate carboxykinase; PPAR, peroxisome proliferator-activated receptor; SREBP, sterol regulatory element-binding protein; VLDL, very-low-density lipoprotein.

While multiple reviews have examined lncRNAs in hepatic metabolism, most focus on a single metabolic pathway or disease entity, leaving a gap in integrated synthesis across cholesterol, triglyceride, and glucose homeostasis and their linked pathological progression. Conceptually, these three metabolic axes are tightly interconnected via shared core transcriptional regulators, such as SREBPs, PPARs, FoxO1, and upstream signaling cascades, including PI3K/protein kinase B (AKT) and mTOR. lncRNAs act as pleiotropic upstream modulators that target these shared nodes at transcriptional, post-transcriptional and epigenetic levels to coordinately shape metabolic homeostasis and reprogramming [14,15]. Therefore, lncRNAs can interact with most cells in vivo, participating in diverse metabolism processes and their associated diseases in humans, such as lipid, glucose, and cholesterol metabolism [16]. Given their widespread involvement in physiological and pathological processes, lncRNAs have attracted substantial research interest as potential diagnostic biomarkers and therapeutic targets. This review provides an updated, systematic synthesis of lncRNA functions across all three hepatic metabolic pathways and their implications in MASLD, hepatocellular carcinoma and type 2 diabetes, describes shared mechanistic pathways, and highlights unresolved mechanistic and translational questions to advance future research (Figure 1).

Relevant studies in this review published over the last ten years were collected through a systematic search of several academic databases, namely, PubMed, Web of Science, Scopus, and Google Scholar. The keyword strategy paired “lncRNA” with metabolic terms such as “hepatic metabolism”, “cholesterol”, “triglyceride”, and “glucose homeostasis”, as well as disease terms including “MASLD”, “hepatocellular carcinoma”, “type 2 diabetes mellitus”, and “gestational diabetes mellitus”, with additional filters applied for study design and language. Preference was given to mechanistic investigations carried out at the in vitro, in vivo, and clinical levels. The collected evidence was then consolidated to explore the role of lncRNAs in maintaining hepatic metabolic balance and in the pathogenesis of related diseases.

## 2. LncRNAs in Lipid Metabolism

Accumulating evidence demonstrates that lncRNAs modulate lipid metabolism by altering the expression of relevant genes, acting at multiple regulatory levels, including intervening in the metabolism of cholesterol or triacylglycerol, and regulating lipid uptake and efflux functions. Their targets comprise key genes and associated regulatory axes or networks. The studies reviewed below were conducted under distinct physiological or pathological conditions, including obesity, insulin resistance, hepatic steatosis, and malignancy, and their findings are interpreted within the corresponding context. Mechanisms observed in disease models should not be assumed to operate identically in the healthy liver.

### 2.1. LncRNAs in Cholesterol Metabolism and Homeostasis

Cholesterol, as a structural and signaling lipid, is fundamental for regulating lipid metabolism [17,18]. Sterol regulatory element binding proteins (SREBPs), master transcriptional regulators of cholesterol and fatty acid synthesis, are controlled by lncRNAs via modulation of their expression [19]. The SREBP family comprises three isoforms, including SREBP-1a, SREBP-1c, and SREBP-2, which drive the synthesis of cholesterol, fatty acids, and triglycerides [20].

Huang et al. revealed that lncARSR is upregulated in hypercholesterolemic patients and C57BL6/j mice fed with a high-cholesterol diet (HCD). In vivo and in vitro experiments have verified that at the gene expression level, lncARSR activates the phosphatidylinositol 3-kinase (PI3K)/AKT pathway to upregulate SREBP-2 and its downstream rate-limiting enzyme HMG-CoA reductase (HMGCR). At the metabolic functional level, this cascade enhances de novo cholesterol biosynthesis and elevates serum and hepatic cholesterol concentrations. SREBP-2 acts as a key transcription factor for HMGCR, the rate-limiting enzyme governing cholesterol synthesis [21,22]. Sallam et al. identified that LeXis, a liver X receptor (LXR)-induced hepatic lncRNA and a regulator of cholesterol homeostasis, lowers serum/hepatic cholesterol in mice on metabolic function by interacting with transcriptional cofactor RALY to inhibit RALY binding to promoters of SREBP-2 [23,24]. lncRNA AT102202 negatively modulates HMGCR expression to reduce cholesterol biosynthesis in cultured human liver (HepG2) hepatocytes [25]. In free fatty acid (FFA)-induced human HepG2 hepatocytes and high-fat-diet-fed C57BL/6J mouse livers, lncRNA growth arrest-specific 5 (GAS5) disrupts hepatic cholesterol metabolism via the miR-29a-3p/NOTCH2 axis. Mechanistically, it enhances cholesterol synthesis by relieving the miR-29a-3p-mediated repression of synthetic enzymes such as HMGCR, resulting in metabolic cholesterol accumulation in the liver [26]. In contrast, lncRNA HOXC-AS1 upregulates homeobox C6 (HOXC6) expression in THP-1 macrophages, and its overexpression partially suppresses oxidized low-density lipoprotein (Ox-LDL)-induced cellular cholesterol accumulation, thereby modulating cholesterol metabolism and mitigating abnormal cholesterol buildup linked to atherosclerosis [27]. Further, lncRNA TRIBAL regulates hepatic cholesterol metabolism primarily by affecting cholesterol synthesis and trafficking. The analysis of human liver tissue cohorts and HepG2 hepatocyte models shows that TRIBAL knockdown downregulates genes involved in cholesterol biosynthesis and cholesterol esterification, without altering cholesterol efflux [28].

Besides cholesterol biosynthesis, cholesterol transport and efflux are critical for lipid homeostasis. It has been reported that Ox-LDL-induced lincRNA-DYNLRB2-2 upregulates G protein-coupled receptor 119 (GPR119) in THP-1 macrophages, which stimulates glucagon-like peptide 1 receptor (GLP-1R) and downstream signaling to enhance ATP-binding cassette transporter A1 (ABCA1)-mediated cholesterol efflux, a finding validated in apoE^−/−^ mice. These observations identify a pivotal lincRNA-DYNLRB2-2/GPR119/GLP-1R/ABCA1 axis governing cholesterol homeostasis [29]. Sallam et al. demonstrated that MeXis, a macrophage-enriched, liver X receptors (LXRs)-β-selective lncRNA, interacts with the transcriptional coactivator DDX17 to increases chromatin accessibility at the Abca1 locus. This interaction promotes ABCA1-dependent cholesterol efflux, and attenuates atherosclerotic lesion progression in mice, with a functionally conserved human ortholog (TCONS_00016111) associated with coronary artery disease risk [30]. lncRNA DAPK1-IT1 modulates cholesterol metabolism via the DAPK1-IT1/hsa-miR-590-3p/ lipoprotein lipase (LPL) axis, which elevates LPL expression, impairs macrophage cholesterol efflux, augments lipid uptake, and disrupts systemic lipid profiles, ultimately driving cholesterol accumulation and atherogenesis in apolipoprotein E-deficient (apoE^−/−^) mice [31]. Elevated lncRNA RP5-833A20.1 expression in THP-1 macrophages blunts cholesterol efflux. This regulatory effect on macrophage cholesterol levels is mediated by the lncRNA-induced upregulation of miR-382-5p, which in turn downregulates nuclear factor IA (NFIA) levels [32]. Lan et al. characterized lnc-HC, a novel hepatocyte-enriched lncRNA, in primary rat hepatocytes and high-cholesterol-diet-fed rat models. lnc-HC is induced by high cholesterol through transcription factor C/EBPβ, and forms a complex with hnRNPA2B1 within the nucleus to bind and destabilize cholesterol 7α-hydroxylase (Cyp7a1) and ABCA1 mRNAs, thereby repressing hepatocytic cholesterol metabolism. The hepatic knockdown of lnc-HC rescues cholesterol metabolic disturbances in vivo [33]. lncRNA MALAT1 is upregulated via DNA hypomethylation in atherosclerotic cardiovascular disease. It activates nuclear factor-κB (NF-κB) signaling, which markedly reduces the expression of cholesterol transporters ABCA1 and ABCG1. Consequently, macrophage cholesterol efflux is strongly suppressed, leading to cholesterol overload, foam cell formation, and accelerated atherosclerosis [34]. lncRNA ZFAS1 is markedly upregulated in ox-LDL-stimulated macrophage foam cells. Acting as a competing endogenous RNA (ceRNA), it sponges and suppresses miR-654-3p activity, leading to increased ADAM10 and RAB22A expression. This axis inhibits macrophage cholesterol efflux, favoring cholesterol accumulation and atherosclerosis progression [35]. lncRNA cyclin-dependent kinase inhibitor 2B antisense RNA 1 (CDKN2B-AS1) promotes macrophage cholesterol efflux by recruiting DNA methyltransferase 1 (DNMT1) to induce the methylation of the A disintegrin and metalloprotease 10 (ADAM10) promoter, which represses ADAM10 expression and reduces inflammatory responses, thereby enhancing cellular cholesterol clearance and the mitigation of atherosclerosis [36]. lncRNA CHROME, a primate-specific lncRNA, supports LXR-driven cholesterol efflux under high-cholesterol conditions. It acts as a sponge to sequester and degrade miR-27b, miR-33a/b and miR-128, thereby relieving their repression of ABCA1 and thereby boosting cholesterol efflux and HDL biogenesis in hepatocyte and macrophages [37,38]. Moreover, lncRNA prostate cancer antigen 3 (PCA3) significantly enhances cholesterol efflux by interacting with miR-140-5p to upregulate the regulatory factor X7 (RFX7), but without RNA immunoprecipitation (RIP) and RNA pull-down assays, which transcriptionally activate ABCA1 expression in macrophages and facilitate reverse cholesterol transport and lipid clearance [39]. By repressing Toll-like receptor 2 (TLR2) to increase ABCA1 abundance, lincRNA DYNLRB2-2 substantially enhances macrophage cholesterol efflux and reduces intracellular cholesterol accumulation and foam cell formation [40]. In THP-1 macrophages, lncRNA NIPA1-SO upregulates ABCA1 and ABCG1 expression via the BMPR2-Smad1/5/8 pathway to stimulate cholesterol efflux. In apoE^−/−^ atherosclerotic animal models, it lowers intracellular cholesterol burden and improves circulating lipid profiles, lowering total cholesterol and LDL-C while raising HDL-C [41]. In mouse primary hepatocytes and C57BL/6J mouse livers, lncRNA Hnf1aos1 knockdown decreases CYP7A1, disrupting cholesterol catabolism. As a compensatory mechanism, ABCA1 and APOB are upregulated to enhance hepatocytes cholesterol efflux [42]. Conversely, lncRNA DANCR regulates macrophage cholesterol metabolism predominantly by suppressing cholesterol efflux and promoting cholesterol uptake rather than altering cholesterol synthesis. It downregulates ABCA1, ABCG1 and scavenger receptor class B type I (SR-BI) to reduce cholesterol efflux, and upregulates CD36 and scavenger receptor class A (SR-A) to enhance cholesterol uptake, driving lipid buildup in macrophages [43]. The DNA methyltransferase 3 beta (DNMT3B)-mediated downregulation of maternally expressed gene 3 (*MEG3*) suppresses ABCA1-dependent cholesterol efflux in endothelial cells via the miR-181a-5p/ABCA1 axis. Notably, *MEG3* functions independently of lncRNA taurine-upregulated gene 1 (*TUG1*), and the two lncRNAs cooperatively restrict cholesterol efflux [44]. lncRNA KCNQ1OT1 represses cholesterol efflux by sequestering miR-452-3p to elevate histone deacetylase 3 (HDAC3), which subsequently downregulates ABCA1 expression and reduces cholesterol efflux from macrophages, promoting macrophage lipid accumulation [45]. lncRNA APAT promotes cholesterol accumulation and suppresses cholesterol efflux in macrophages both in vitro and in vivo. It upregulates the lipid phagocytosis gene *CD36* while repressing cholesterol efflux genes encoding scavenger receptor B1 (SR-B1) and ABCG1 [46].

This study demonstrates that the knockdown of lncRNA GAS5 inhibits the progression of atherosclerosis in ApoE-deficient mice by reducing the enhancer of zeste homolog 2 (EZH2)-mediated transcriptional repression of ABCA1 via histone methylation in bone marrow-derived macrophages, thereby promoting reverse cholesterol transport and inhibiting lipid accumulation in foam cell [47]. In human HepG2 hepatocytes and mouse liver tissues, lncRNA APOA1-AS downregulates the expression of Apolipoprotein A1 (APOA1), the major structural component of HDL, and its neighboring cluster genes *APOC3/APOA4* by recruiting lysine (K)-specific demethylase 1 (LSD1) and Polycomb repressive complex 2 (PRC2) to establish repressive histone modifications, thereby impairing reverse cholesterol transport. Conversely, its knockdown upregulates these apolipoproteins to enhance HDL-mediated cholesterol efflux and hepatic cholesterol clearance [48]. In human HepG2 hepatocytes and murine cholestatic liver fibrosis models, lncRNA H19 is induced by bile acids and interacts with hepatic zinc finger E-box-binding homeobox 1 (ZEB1) to block its binding to the epithelial cell adhesion molecule (EpCAM) promoter, thereby activating EpCAM expression and disrupting bile acid transport homeostasis, a critical route for cholesterol elimination, indirectly altering cholesterol metabolism and accelerating cholestatic liver fibrosis [49]. Similarly, lncRNA H19, whose expression is de-repressed following the Bcl2-driven, caspase-8-dependent degradation of small heterodimer partner (SHP), disrupts bile acid homeostasis by compromising bile acids synthesis and transport, which indirectly perturbs cholesterol transport [50]. In mouse primary hepatocytes and cholestatic mouse models, lncRNA MEG3 acts as a scaffold to recruit PTBP1 to Shp mRNA, accelerating its degradation and relieving the Shp-mediated transcriptional repression of Cyp7a1 and sterol 12α-hydroxylase (Cyp8b1), the key enzymes governing cholesterol into bile acids. This cascade ultimately accelerates hepatic cholesterol synthesis into bile acids and disrupts cholesterol homeostasis coupled with bile acid balance [51]. lncRNA APOA4-AS, a positive regulator of APOA4, interacts with mRNA-stabilizing protein HuR to maintain APOA4 expression. Its knockdown in ob/ob mice lowers hepatic APOA4 levels and markedly reduces plasma triglyceride and total cholesterol concentrations, with no change in hepatic triglyceride storage, linking this lncRNA to systemic lipid homeostasis in obesity and fatty liver disease [52]. lncRNA BM450697, an antisense transcript to low-density lipoprotein receptor (LDLR) promoter, represses LDLR transcription through DNA–RNA hybrid formation and transcriptional interference, reducing hepatic LDL uptake and raising circulating cholesterol level, while its silencing de-represses LDLR expression and enhances LDL cholesterol uptake in human liver cells, supporting its potential as a therapeutic target for hypercholesterolemia [53]. The overexpression of the long non-coding RNA RP1-13D10.2 in Huh7 and HepG2 cells boosts hepatic LDL cholesterol clearance by activating LDLR transcription, modulates statin-induced cholesterol-lowering efficacy, and is sterol-regulated to maintain cholesterol homeostasis, representing a novel modulator of cholesterol metabolism and a potential determinant of inter-individual statin efficacy [54]. Additionally, macrophage cholesterol efflux is the core link of reverse cholesterol transport, and peripheral cholesterol ultimately flows back to the liver through HDL for metabolic clearance, directly affecting liver cholesterol homeostasis. This also explains why several experiments utilized THP-1 macrophages.

Taken together, lncRNAs regulate hepatic cholesterol homeostasis through three recurring mechanistic paradigms with differing evidence strengths. The transcriptional and epigenetic control of the SREBP-2/HMGCR biosynthetic axis is the most well-established, with lncRNAs such as LeXis and lncARSR validated in both human hepatocytes and in vivo murine models. The regulation of mRNA stability via RNA-binding proteins hnRNPA2B1 and IGF2BP1 is biochemically supported for several transcripts, but its physiological relevance in a non-diseased liver remains partially speculative. The ceRNA sponge mechanism is the most widely reported mode for cholesterol efflux regulators, yet most studies rely on overexpression systems, and the endogenous stoichiometric feasibility of many proposed ceRNA interactions in hepatocytes remains controversially.

**Figure 2:**
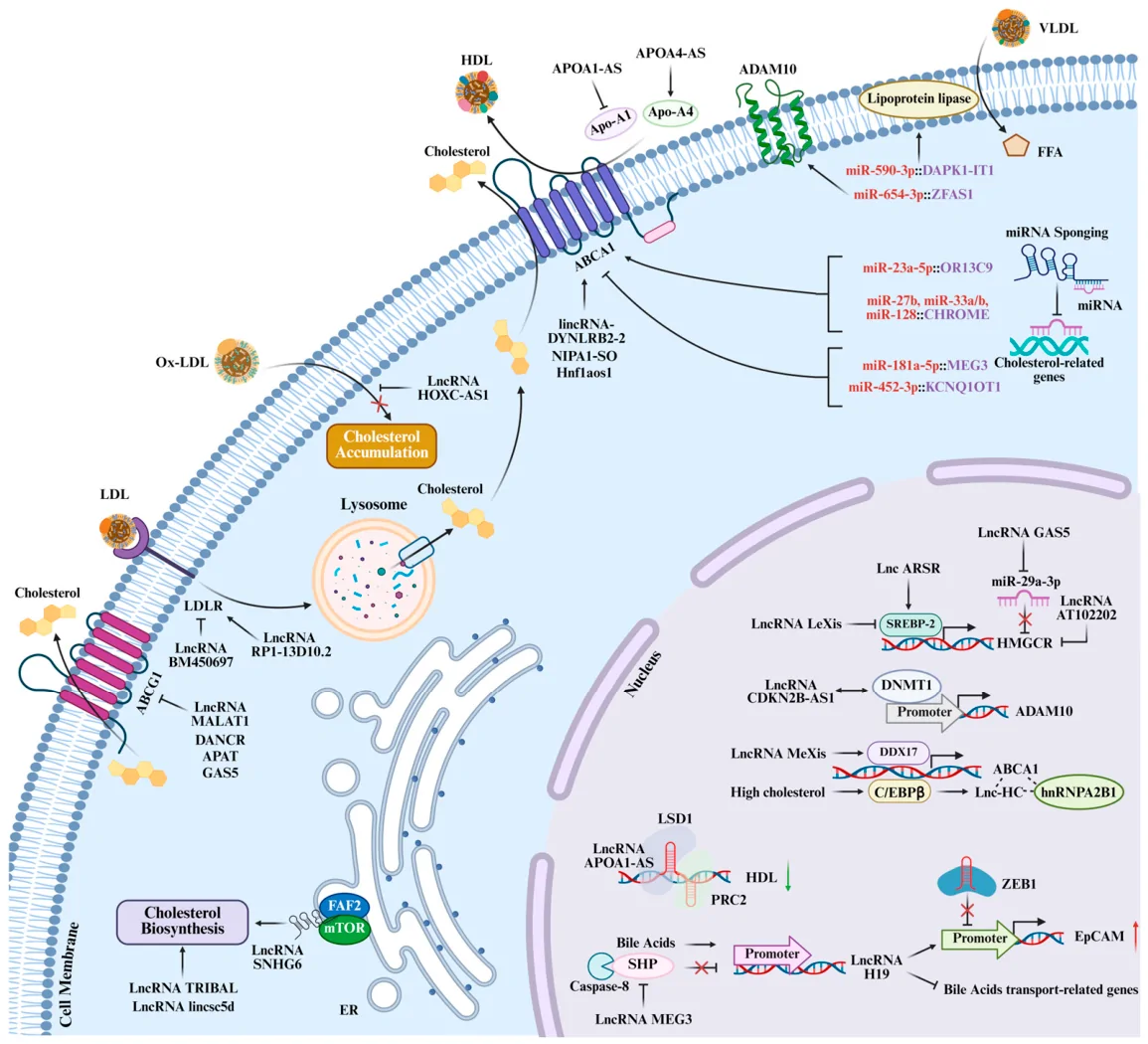
Diagram illustrating the regulatory roles of lncRNAs in cholesterol metabolism. The diagram depicts the multiple layers of regulation exerted by lncRNAs over cholesterol metabolic networks. At the plasma membrane, lncRNAs modulate cholesterol uptake by LDLR and cholesterol efflux mediated by ABCA1, alongside the lipoprotein metabolism of LDL, oxidized LDL, HDL and VLDL. Within the endoplasmic reticulum, lncRNAs govern cholesterol biosynthesis. A subset of cytoplasmic lncRNAs restrain miRNA activity to relieve the miRNA-mediated repression of cholesterol-associated genes. Nuclear lncRNAs govern gene expression via interactions with transcription factors and epigenetic complexes. Multiple lncRNAs are involved in bile acid transport and metabolism. Black sharp arrows represent induction or progression, while black flat-headed arrows represent inhibitory effects. The green downward arrow represents the downregulation of expression, while the red upward arrow represents the upregulation of expression. Cross symbols denote prevention or impediment of target processes. ABCA1, ATP-binding cassette transporter A1; ADAM10, ADAM metallopeptidase domain 10; APOA1, apolipoprotein A1; APOA4, apolipoprotein A4; CDKN2B-AS1, CDKN2B antisense RNA 1; DNMT1, DNA methyltransferase 1; EAF2, ELL-associated factor 2; EpCAM, epithelial cell adhesion molecule; ER, endoplasmic reticulum; FFA, free fatty acid; GAS5, growth arrest specific 5; HDL, high-density lipoprotein; HMGCR, 3-hydroxy-3-methylglutaryl-coenzyme A reductase; HNF1AOS1, HNF1A antisense RNA 1; HOXC-AS1, HOXC cluster antisense RNA 1; hnRNPA2B1, heterogeneous nuclear ribonucleoprotein A2/B1; LDL, low-density lipoprotein; LDLR, low-density lipoprotein receptor; lnc, long non-coding; LSD1, lysine specific demethylase 1; MALAT1, metastasis-associated lung adenocarcinoma transcript 1; MEG3, maternally expressed gene 3; mTOR, mechanistic target of rapamycin kinase; Ox-LDL, oxidized low-density lipoprotein; PRC2, Polycomb repressive complex 2; SHP, small heterodimer partner; SREBP-2, sterol regulatory element-binding protein 2; VLDL, very-low-density lipoprotein; ZEB1, zinc finger E-box binding homeobox 1.

The overall mechanisms and roles of lncRNA in cholesterol metabolism are shown in Figure 2 and Table 1.

### 2.2. LncRNAs in Triacylglycerol Metabolism and Homeostasis

Liu et al. found in mouse primary hepatocytes and high-fat, high-sucrose (HFHS)-diet-fed C57BL/6J mice that lncRNA H19 interacts with Polypyrimidine tract-binding protein 1 (PTBP1) to form a feedforward regulatory circuit that drives hepatic triacylglycerol (TG) accumulation by stabilizing SREBP-1c mRNA, facilitating SREBP1 protein cleavage and nuclear translocation, and amplifying its transcriptional activity. This circuit is induced by fatty acids and the HFHS diet, leading to widespread increases in hepatic TG species and steatosis in mice [55]. The analysis of serum samples from MASLD patients and FFA-induced human L02 hepatocytes shows that lncRNA H19 is upregulated in hepatic steatosis, promotes triglyceride synthesis and storage by activating lipogenic transcription factor MLXIPL and the mTORC1 signaling cascade, upregulates lipogenic genes, and shifts hepatic lipid balance toward lipid accumulation [56]. Zhao et al. reported that lncRNA Blnc1, abundantly expressed in the livers of obese and MASLD mice with elevated triacylglycerol levels, assembles a ribonucleoprotein complex with endothelial differentiation-related factor 1 (EDF1) and LXRα. This complex activates SREBP-1c and hepatic lipogenic genes, thereby stimulating de novo lipogenesis and increasing triacylglycerol pools in the liver and circulation [57]. Yan et al. revealed in HepG2 hepatocytes and high-fat-diet-fed mouse livers that lncRNA MALAT1 directly binds to nuclear SREBP-1c to increase its protein stability by blocking ubiquitin-dependent degradation, thereby upregulating SREBP-1c-targeted lipogenic genes, promoting hepatic triacylglycerol and cholesterol accumulation, and triggering hepatic steatosis and insulin resistance [58]. In high-glucose-treated HepG2 hepatocytes and liver tissues from mice with diabetes complicated by MASLD, lncRNA Gm10804 promotes hepatic triglyceride accumulation by upregulating SREBP-1c/FAS to boost fatty acid synthesis to further fuel TG buildup [59]. Similarly, in human L02 hepatocytes and MASLD mouse liver tissues, lncRNA H19 restrains miR-130a to upregulate peroxisome-proliferator-activated receptor gamma (PPARγ), upregulating lipogenic genes including fatty acid synthase (*FASN*), acetyl-CoA carboxylase alpha (*ACACA*) and stearoyl-CoA desaturase 1 (*SCD1*) to boost hepatic triglyceride synthesis and lipid droplet deposition in hepatocytes [60]. Moreover, lnc19959.2 promotes hepatic triglyceride secretion by inducing ubiquitination of the transcriptional repressor Purb to elevate ApoA4 abundance. It also binds hnRNPA2B1 to downregulate Cpt1a, Gpam and Tm7sf2, and restrain fatty acid oxidation and TG synthesis in rat livers [61]. In SRA-knockout mouse livers and human hepatocyte models, lncRNA SRA suppresses hepatic triglyceride lipolysis predominantly by repressing the forkhead box protein O1 (FoxO1)/PPARγ-driven of adipose triacylglycerol lipase (ATGL), which reduces ATGL-mediated triglyceride lipolysis and drives hepatic TG accumulation [62].

Moreover, lncRNA PU.1 AS in mice and liver cells physically interacts with EZH2 to block EZH2 recruitment to the Sirtuin 6 (Sirt6) promoter, thereby attenuating Sirt6 expression, downregulating SREBP-1c protein levels, and reducing triacylglycerol synthesis and lipid deposition in hepatocytes [63]. Similarly, Li et al. identified lncHR1, a novel human-specific lncRNA that acts as a negative regulator of SREBP-1c. lncHR1 overexpression represses SREBP-1c and its downstream target lipogenic gene *FASN*, diminishing oleic acid-induced triacylglycerol (TG) buildup and lipid droplet formation in hepatocytes [64]. In murine AML12 hepatocytes and high-fat-diet-fed mouse livers, lnc027912 increases the abundance of phosphorylated AMPKα and decreases mTOR phosphorylation, thereby lowering nuclear SREBP-1c expression and suppressing the SREBP-1c promoter. These combined effects dampen lipogenesis and mitigate triglyceride accumulation both in vitro and in vivo [65]. In human HepG2 hepatocytes and MASLD mouse models, lncRNA MEG3 reduces intracellular and hepatic triglyceride deposition by inhibiting triglyceride biosynthesis. It represses the expression of lipogenic genes including *CD36, FASN, ACACA, SCD1*, and *SREBP-1c* to curb de novo triglyceride production and fatty acid uptake via the EZH2/SIRT6 axis, thereby alleviating triglyceride-associated hepatic steatosis [66]. In whole-body NRON-knockout mice, lncRNA NRON deficiency reduces hepatic triglyceride accumulation by inhibiting lipogenic gene expression through the FGF21/AMPK/SREBP1 pathway, thereby blunting hepatic triglyceride synthesis. In adipose tissue, NRON depletion stimulates triglyceride hydrolysis and facilitates triacylglycerol (TAG)/FA cycling to increase fatty acid mobilization. These integrated actions lower systemic and hepatic triglyceride levels and protect against diet-induced hepatic steatosis [67]. In supplement, FFAs released by lipolysis of adipose tissue enter the liver through the blood circulation and are an important substrate source for triglyceride synthesis in the liver. Thereby, the regulatory pathway of adipose tissue directly affects the homeostasis of liver lipid metabolism. lncRNA lnc-HC, upregulated in FFA-treated hepatocytes and high-fat, high-cholesterol-diet (HCD)-fed rat livers, downregulates PPARγ expression by elevating miR-130b-3p expression at both transcriptional and post-transcriptional levels, thereby suppressing hepatocytic triglyceride (TG) synthesis, lipid droplet accumulation, and hepatic steatosis [68]. lnc-LLMA is a nucleus-enriched, liver-specific lncRNA that promotes triglyceride trafficking and secretion by upregulating microsomal triglyceride transfer protein (MTTP), while restraining triglyceride synthesis via downregulating SREBP1 and G6Pase. It directly interacts with MTTP and glycogen synthase 2 (Gys2) to regulate triglyceride metabolism, and the overexpression of either protein can rescue the triglyceride-lowering effect of lnc-LLMA [69]. Meanwhile, in human Huh7 hepatocytes and high-fat-diet (HFD)-fed mouse liver models, lncRP11-675F6 associates with hexokinase 1 (HK1) to govern hepatic triglyceride trafficking. Its deficiency activates autophagy, which degrades proteins required for very-low-density lipoprotein (VLDL) assembly, including apolipoprotein 100 (ApoB100) and MTTP. This disruption impedes liver TG secretion and results in intracellular lipid buildup, whereas intact lncRP11-675F6 limits excessive autophagy and facilitates VLDL formation and TG export to mitigate HFD-triggered fatty liver [70].

Liver-enriched lncRNA LSTR interacts with TDP-43 to regulate Cyp8b1 and shape bile acid composition, inhibiting farnesoid X receptor (FXR)/apoC2/LPL signaling and reducing triglyceride clearance. Its depletion amplifies this cascade, accelerating plasma triglyceride clearance in mice [71]. lncRNA GM47544 reduces intracellular and plasma triglyceride levels by promoting the K48-linked ubiquitination and proteasomal degradation of APOC3 at lysine 79, which results in increasing triglyceride clearance, thereby alleviating hepatic steatosis and atherosclerosis [72]. AdipoQ AS, an adiponectin antisense lncRNA, translocates from the nucleus to the cytoplasm and base-pairs with AdipoQ mRNA to form an RNA duplex, blocking AdipoQ protein translation and reducing adiponectin production. In high-fat-diet-fed mice, this lncRNA lowers triglyceride synthesis and accumulation in the liver, decreases hepatic lipid droplet formation, and ultimately ameliorates hepatic steatosis to restore lipid metabolism balance [73].

For hepatic triglyceride metabolism, nearly all identified lncRNAs converge on the central SREBP-1c lipogenic cascade, falling into two main regulatory modes. The direct modulation of SREBP-1c protein stability, cleavage, or nuclear translocation, like H19 and MALAT1, represents a well-established mechanism consistently reproduced across multiple in vivo and in vitro systems. The ceRNA-mediated regulation of upstream PI3K/AKT/mTOR signaling is extensively documented, but with highly variable rigor; a subset of axes (e.g., H19/miR-130a) have in vivo loss-of-function validation, while many others remain correlative observations from cell-based overexpression assays, with unconfirmed physiological relevance.

## 3. LncRNAs in Glucose Metabolism in Liver

**Figure 3:**
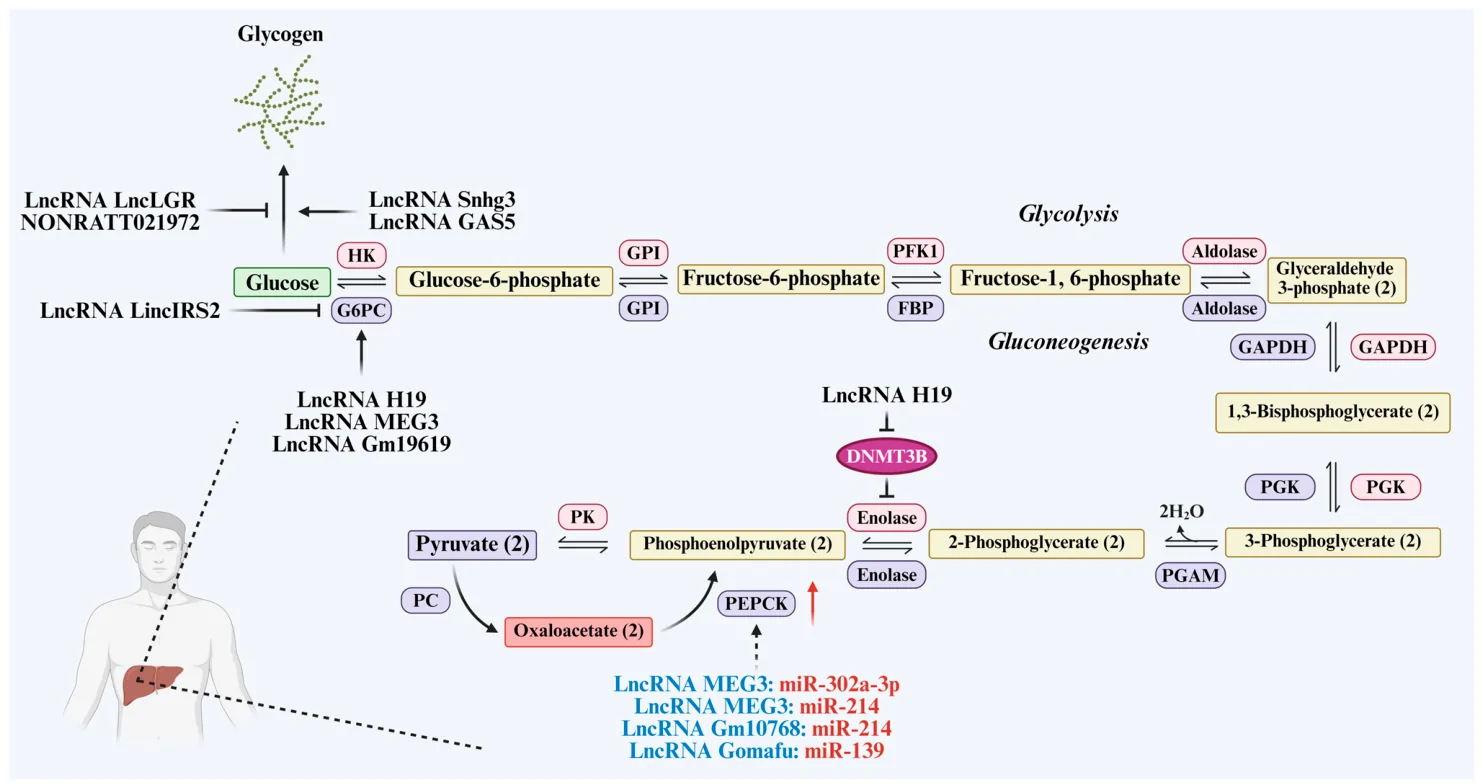
The modulation of lncRNAs in hepatic glucose metabolism. This schematic summarizes lncRNA-dependent regulatory mechanisms governing hepatic gluconeogenesis, glycogen metabolism and glycolysis. Multiple lncRNAs, including H19, MEG3 and Gm19619, regulate key rate-limiting gluconeogenic enzymes such as G6Pase and PEPCK. lncRNA H19 suppresses DNMT3B to modulate enolase activity, while MEG3, Gm10768 and Gomafu restrain miRNA function to relieve the microRNA-mediated repression of PEPCK. Additional lncRNAs (Snhg3, GAS5, lncLGR, LincIRS2) control glycogen turnover and glucose homeostasis, balancing gluconeogenic and glycolytic flux within hepatocytes. Black sharp arrows represent induction or progression, while black flat headed arrows represent inhibitory effects. The green downward arrow represents downregulation of expression, while the red upward arrow represents upregulation of expression. The dashed line represents indirect effects, with other signal components in between. DNMT3B, DNA methyltransferase 3 beta; FBP, fructose bisphosphatase; G6Pase, glucose-6-phosphatase; GAPDH, glyceraldehyde-3-phosphate dehydrogenase; GPI, glucose-6-phosphate isomerase; HK, hexokinase; lncRNA, long non-coding RNA; PC, pyruvate carboxylase; PEPCK, phosphoenolpyruvate carboxykinase; PFKI, phosphofructokinase 1; PGAM, phosphoglycerate mutase; PGK, phosphoglycerate kinase; PK, pyruvate kinase.

The liver is involved in the glucose metabolism, including glycogen synthesis through glycogenesis, the breakdown of glycogen through glycogenolysis to release glucose into blood, and the synthesis of glucose via gluconeogenesis (Figure 3). As in lipid metabolism, the glucose-related LncRNA mechanisms discussed below span both normal homeostasis and diseased conditions, and their effects are tied to the prevailing nutritional and hormonal state.

### 3.1. Regulations of lncRNAs on Glycogen Metabolism

For glycogen metabolism, lncLGR, a hepatic glucokinase (GCK) repressor, is induced by fasting in mouse primary hepatocytes and C57BL/6J mouse livers to recruit heterogenous nuclear ribonucleoprotein L (hnRNPL) to the GCK promoter to repress GCK transcription. Reduced GCK expression diminishes glucose-6-phosphate (G6P) production, restricting hepatic glycogen synthesis [74]. In liver tissues from type 2 diabetic rats and BRL rat hepatocyte lines, upregulated lncRNA NONRATT021972 impairs AKT activation in diabetic liver, represses glucokinase, and reduces glucose phosphorylation and glycogen synthesis. Its knockdown increases phosphorylated AKT, suppresses active glycogen synthase kinase-3β (GSK-3β), and restores glucokinase activity to promote hepatic glycogen formation and lower circulating glucose levels [75]. Conversely, lncRNA Snhg3 predominantly regulates hepatic glycogenesis in mouse primary hepatocytes and C57BL/6J mouse livers by inducing chromatin remodeling and elevating AKT phosphorylation, which upregulates protein phosphatase 1 regulatory subunit 3B (PPP1R3B) expression to boost glycogen synthesis and storage in hepatocytes, thereby improving glucose tolerance and sustaining hepatic glucose homeostasis [76]. In diet-induced obese diabetic mouse livers and mouse hepatocyte models, lncRNA GAS5 markedly raises the expression of Gys2, which accelerates glycogen synthesis and facilitates glucose storage [77].

### 3.2. Regulations of lncRNAs on Gluconeogenesis

For gluconeogenesis, lncRNA H19 depletion elevates hepatic VDAC1 expression, promotes the phosphorylation of c-Jun N-terminal kinase (JNK) and insulin receptor substrate 1 (IRS1), and inhibits insulin-induced AKT activation in mice, ultimately enhancing gluconeogenesis and disturbing liver glucose homeostasis [78]. In mouse primary hepatocytes and db/db diabetic mouse models, H19 facilitates hepatic gluconeogenesis by binding and inhibiting S-adenosylho mocysteine hydrolase (SAHH). This interaction reduces methylation at the hepatocyte nuclear factor 4α (Hnf4a) promoter, and elevates Hnf4a and downstream gluconeogenic enzymes PEPCK/G6PC to boost hepatic glucose production and trigger hyperglycemia [79]. In addition to H19, upregulated lncRNA MEG3 elevates FoxO1 in human HepG2 hepatocytes and diabetic rat livers, upregulating core gluconeogenic enzymes, like G6PC and PEPCK, and enhancing hepatic gluconeogenesis-derived hepatic glucose production while inhibiting insulin-triggered hepatic glycogen synthesis [80]. Further, in mouse primary hepatocytes and db/db mouse liver tissues, cAMP-response element binding protein (CREB)-induced MEG3 acts as a ceRNA to sponge miR-302a-3p to elevate CREB-regulated transcriptional coactivator 2 (CRTC2) expression and activate the PPARγ coactivator-1α (PGC-1α)-PEPCK/G6PC pathway, inducing gluconeogenesis gene expression and amplifying hepatic gluconeogenesis [81]. As a ceRNA sponging miR-214 in HepG2 hepatocytes and diabetic mouse livers, MEG3 strengthens the activating transcription factor 4 (ATF4)-FoxO1 axis to induce core gluconeogenic enzymes PEPCK and G6Pase, accelerating hepatic gluconeogenesis and suppressing insulin-dependent glycogen synthesis [82]. Similarly, lncRNA Gm10768 competitively absorbs miR-214 to upregulate ATF4 and stimulate gluconeogenesis in mouse primary hepatocytes and db/db diabetic mouse livers [83]. lncRNA Gm19619 interacts with FoxO1 and increases its abundance, which elevates gluconeogenic genes *G6PC* and *Pck1*, promoting hepatic gluconeogenesis and leading to impaired insulin sensitivity in murine AML12 hepatocytes and mouse liver tissues [84]. Furthermore, lncRNA Gomafu sponges miR-139 to relieve miR-mediated repression of FoxO1, increasing the expression of PEPCK and G6Pase and amplifying hepatic gluconeogenesis in HepG2 hepatocytes and diabetic mouse liver models [85]. Similarly, lncRNA Gm10804 is upregulated in high glucose-treated hepatocytes and in the livers of mice with diabetes complicated by MASLD, which elevates gluconeogenic enzymes PEPCK and G6Pase to drive hepatic glucose production, and the hepatic knockdown of Gm10804 concomitantly restrains gluconeogenesis and alleviates steatosis in vivo [59]. Furthermore, the MAFG-LincIRS2 axis regulates hepatic glucose metabolism. Nutrient excess and obesity induce MAFG overexpression in livers and primary hepatocytes from obese mice, which represses lncRNA LincIRS2. LincIRS2 depletion significantly upregulates gluconeogenic genes, such as *PEPCK* and *G6PC*, enhancing hepatic gluconeogenesis and driving hyperglycemia and insulin resistance [86]. Shcherbinina et al. found that lncRNA Falcor/LL35 knockdown markedly upregulates the expression of key genes governing gluconeogenesis, including *G6PC* and *Pck1* in murine AML12 cells, indicating enhanced gluconeogenic activity [87].

Conversely, lncRNA H19 restrains hepatic gluconeogenesis in vivo by blocking the p53 recruitment to the FoxO1 promoter to lower FoxO1 level, downregulating gluconeogenic enzymes and limiting excessive hepatic glucose generation [88]. Similarly, H19 maintains intact hepatic insulin–AKT signaling in HepG2 cells, restricting FoxO1 nuclear translocation to inhibit gluconeogenic enzyme expression and curb hepatic glucose synthesis [89]. lncRNA lncSHGL suppresses hepatic gluconeogenesis in the obese mouse liver via recruiting heterogenerous nuclear ribonucleoprotein A1 (hnRNP1) to increase CALM and calmodulin translation levels. This cascade activates PI3K/AKT phosphorylation and inhibits the mTOR/SREBP-1c pathway, repressing gluconeogenic genes’ expression and attenuating hyperglycemia in obese mice [90]. Shen et al. found that a deficiency of lncRNA betaine-homocysteine methyltransferase-antisense (Bhmt-AS) suppresses hepatic gluconeogenesis in primary hepatocytes by regulating Bhmt expression and reduces liver glucose production and gluconeogenic gene expression in vivo [91]. In addition, lncRNA Metastasis-associated lung adenocarcinoma transcript 1 (MALAT1) downregulates gluconeogenic enzymes by elevating the level of metabolic transcription factor TCF7L2, which interacts with the promoter of G6PC and PCK1 in HepG2 and FLC4 cells, thereby repressing gluconeogenesis [92]. The metformin-mediated downregulation of NONMMUT031874.2 enhances miR-7054-5p expression and decreases Socs3 expression, which inhibits PEPCK to repress gluconeogenesis [93].

### 3.3. Regulations of lncRNAs on Glycolysis

For glycolysis, murine lncRNA Falcor/LL35, a putative functional analog of human lncRNA DEANR1/linc00261, modulates hepatic glucose metabolism in vitro and in vivo. LL35 knockdown significantly suppresses glycolysis, as reflected by the reduced extracellular acidification rate together with lowered glycolytic capacity and glycolytic reserve in normal murine AML12 hepatocytes [87]. lncRNA H19 modulates hepatic glucose metabolism via the H19–DNMT3B epigenetic axis. H19 downregulation elevates DNMT3B expression, which induces promoter hypermethylation and silences the two glycolytic genes Hk2 and Eno3. This suppresses glycolytic enzyme activity, reduces glycolytic flux and diminishes cellular ATP production in diabetic mouse models and hepatocytes [94]. lncRNA Tug1 functions as a ceRNA to bind and inhibit miR-1934-3p in hepatocytes to suppress excessive hepatic glycolysis and attenuate intracellular lipid deposition [95]. lncRNA lncLGR is a liver-specific lncRNA induced under fasting conditions. It associates with hnRNPL to inhibit glucokinase expression, thereby suppressing hepatic glycolysis and reducing glycogen storage [74].

Collectively, lncRNAs regulating hepatic glucose metabolism mainly target two core modules, the gluconeogenic transcription factor network FoxO1 and HNF4α, and the insulin-PI3K/AKT signaling pathway. The H19-DNMT3B-mediated epigenetic silencing of glycolytic genes is a well-established mechanism, with consistent validation in rodent diabetic models. In contrast, the FoxO1-centered ceRNA-regulatory pattern is reported in multiple lncRNAs, but most supporting data come from murine cells and animal models, whose functional conservation in the human liver remains largely speculative. The overall mechanisms and roles of lncRNA in glucose metabolism are shown in Table 2.

## 4. Clinical Roles and Mechanisms of lncRNAs for Glucose and Lipid Metabolism-Related Diseases in Liver

### 4.1. LncRNA in Hepatocellular Carcinoma

Hepatocellular carcinoma (HCC) remains a primary driver of cancer-related disease and fatalities. Based on GLOBOCAN 2022 estimates, it takes the fifth place in male populations and the ninth place in female populations, and was the third leading cause of cancer-related death, especially occurring in East and South-East Asia and North Africa, placing great human health-, social- and economy-related burdens on the world [96,97,98]. The progression from MASLD to MASH, fibrosis and ultimately HCC forms a continuous pathological cascade. Hepatic lipid accumulation and lipotoxicity trigger chronic inflammation and oxidative stress, which drive aberrant metabolic reprogramming and eventual malignant transformation. lncRNAs regulate hepatic lipid and glucose homeostasis in early metabolic dysfunction, modulate inflammatory and fibrogenic signaling in progressive disease, and sustain glycolytic reprogramming in established HCC. The following section details lncRNA-mediated metabolic alterations in HCC and MASLD. In addition, the metabolic reprogramming of glucose metabolism yields sufficient ATP to support robust proliferation and an invasive capacity in HCC, and this process has attracted attention as a promising therapeutic target for tumors [99].

**Figure 4:**
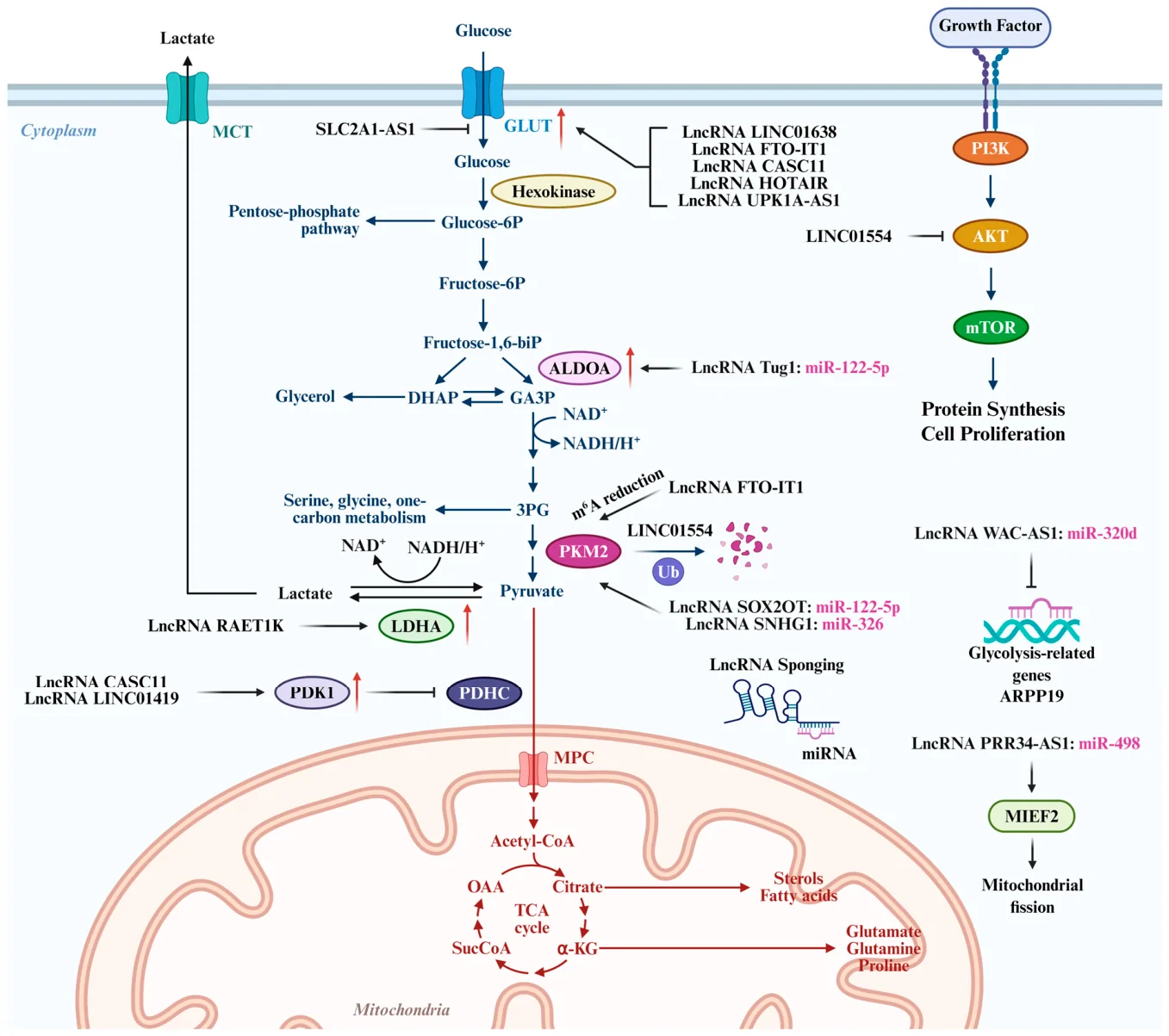
Overview of the lncRNAs-driven metabolic reprogramming of the Warburg effect in hepatocellular carcinoma. This figure uncovers lncRNA-related regulation to aerobic glycolysis or the Warburg effect in HCC cells. Multiple lncRNAs modulate glucose uptake through GLUT transporters and govern the activity of core glycolytic rate-limiting enzymes, including HK, ALDOA and PKM2. Some lncRNAs act as ceRNA to sponge miRNA to relieve the repression of glycolysis-related genes, whereas others regulate the PI3K/AKT/mTOR axis to promote glycolysis and cell proliferation. lncRNAs also modulate lactate production and efflux via LDHA and MCT, and alter pyruvate mitochondrial import through PDK1/PDHC. Black sharp arrows represent induction or progression, while black flat headed arrows represent inhibitory effects. The green downward arrow represents the downregulation of expression, while the red upward arrow represents the upregulation of expression. α-KG, α-ketoglutarate; ALDOA, aldolase A; DHAP, dihydroxyacetone phosphate; FTO-IT1, FTO intronic transcript 1; G3P, glyceraldehyde 3-phosphate; GLUT, glucose transporter; HK, hexokinase; LDHA, lactate dehydrogenase A; MCT, monocarboxylate transporter; MIEF2, mitochondrial elongation factor 2; MPC, mitochondrial pyruvate carrier; mTOR, mechanistic target of rapamycin kinase; OAA, oxaloacetate; PDHC, pyruvate dehydrogenase complex; PDK1, pyruvate dehydrogenase kinase 1; PI3K, phosphatidylinositol 3-kinase; PKM2, pyruvate kinase M2; SucCoA, succinyl-CoA; TCA, tricarboxylic acid; 3PG, 3-phosphoglycerate.

Previous studies have explored lncRNA-dependent glucose metabolic remodeling in clinical HCC specimens (Figure 4). For instances, Chen et al. enrolled 74 patients with HCC and found that lncRNA LINC01638 is highly upregulated in HCC, which elevates the expression of GLUT1 to boost glucose uptake and accelerate hepatocellular carcinoma cell proliferation [100]. In a cohort of 167 primary HCC clinical specimens, Zheng et al. observed that lncRNA LINC01554 is frequently downregulated in tumor tissues relative to matched peritumoral liver tissues. Subsequent functional validation in cultured HCC cell lines demonstrated that LINC01554 exerts a tumor-suppressive effect by promoting the ubiquitin-dependent degradation of pyruvate kinase M2 (PKM2) and inhibiting the AKT/mTOR signaling cascade, which ultimately curbs the Warburg effect in HCC cells [101]. By analyzing 92 paired human HCC and matched peritumoral liver tissues, Wang et al. demonstrated that lncRNA FTO-IT1 is abundantly expressed in HCC. It facilitates HCC cell proliferation and aerobic glycolysis by reducing FTO-mediated m^6^A (N^6^-methyladenosine) modification on GLUT1, PKM2 and MYC proto-oncogene (c-Myc) mRNAs, stabilizing these transcripts and amplifying glycolytic flux. Accordingly, FTO-IT1 represents a candidate prognostic marker and therapeutic target for HCC [102]. Similarly, lncRNA miR4458HG promotes HCC glycolysis via the IGF2BP2 (a key RNA m^6^A reader)/Hk2/GLUT1 axis by tuning m^6^A modification, and drives tumor progression both in vitro and in a cohort of clinical 128 HCC tissues [103]. lncRNA-SOX2OT is upregulated in HCC tissues collected from 121 patients. It enhances glycolysis via sponging miR-122-5p to upregulate PKM2, thereby promoting HCC metastasis [104].

lncRNA SNHG1 is transcriptionally induced by the E2F1 transcription factor. It restrains miR-326 activity to relieve the miR-dependent suppression of PKM2, thereby increasing PKM2 expression and stimulating glycolysis and HCC cell proliferation using 30 paired HCC and adjacent normal tissues [105]. Meanwhile, lncRNA CASC11 recruits the protein EIF4A3 to stabilize E2F1 mRNA, which upregulates glycolytic proteins GLUT1 and Pyruvate dehydrogenase kinase 1 (PDK1), enhancing glucose uptake and lactate production in a set of 78 HCC tissue specimens [106]. The analysis of multiple clinical datasets including The Cancer Genome Atlas (TCGA) revealed that lncRNA LINC01419 is highly expressed in HCC tumor tissues and correlates with aggressive disease phenotypes. Mechanistic experiments in the HCC cell lines showed that LINC01419 recruits Y-box binding protein 1 (YBX1) to stabilize pyruvate dehydrogenase kinase 1 (PDK1) mRNA, which enhances aerobic glycolysis and drives malignant progression. Furthermore, in vivo intervention in BALB/c nude mouse xenograft models confirmed that the targeted knockdown of LINC01419 exerts significant anti-tumor effects [107]. The analysis of 73 paired HCC and peritumoral tissues revealed that the overexpressed lncRNA Ftx activates the PPARγ pathway to enhance aerobic glycolysis and the malignant progression of HCC cells [108]. With 38 paired HCC and peritumoral tissues, Xu et al. revealed that upregulated lncRNA CERS6-AS1 restrains miR-30b-3p activity to facilitate the MDM2-driven ubiquitination of the tumor suppressor p53, thereby amplifying aerobic glycolysis and malignant progression in HCC cells [109]. Similarly, Xia et al. analyzed 62 clinical HCC specimens and transcriptomic datasets, and found that lncRNA WAC-AS1 acts as a ceRNA to sponge miR-320d and upregulate glycolysis-related gene cAMP-regulated phosphoprotein 19 (ARPP19), which enhances aerobic glycolysis and favors malignancy progression [110]. The highly abundant lncRNA PRR34-AS1 restrains miR-498 activity and elevates mitochondrial elongation factor 2 (MIEF2) abundance to promote mitochondrial fission and aerobic glycolysis [111]. Hu et al. exhibited that lncRNA homeobox transcript antisense RNA (HOTAIR) sponges to bind and inhibit miR-130a-3p, which targets and downregulates hypoxia inducible factor 1 alpha (HIF1A), thereby increasing Hk2 and GLUT1 expression and promoting hypoxia-induced glycolysis [112]. In addition, lncRNA RAET1K is activated by HIF1A under hypoxic conditions to suppress miR-100-5p expression, raising lactate dehydrogenase A (LDHA) levels, as observed in 66 clinical HCC specimens [113]. In a cohort of 33 paired HCC samples and non-tumor tissues, lncRNA SLC2A1-AS1 binds transketolase and STAT3 to repress the transcription of *FOXM1* and *GLUT1*. This effect blunts GLUT1 function, inhibits glycolysis and halts HCC progression [114]. Lu et al. utilized 120 HCC patient samples and multiple models to show that cancer-associated fibroblasts (CAFs)-derived exosomal lncRNA TUG1 promotes HCC glycolysis via the miR-524-5p/ sine oculis homeobox homolog 1 (SIX1) regulatory axis [115].

At the cellular and molecular biology level, Zhang et al. showed that lncRNA UPK1A-AS1 enhances glucose glycolysis in HCC by stabilizing the HIF-1α protein to activate the transcription of glycolytic genes including GLUT1, Hk1, Hk2 and phosphoglycerate kinase 1 (PGK1) in the human HCC cell line MHCC-97H [116]. lncRNA Tug1, highly expressed in the HepG2 cell line, sponges and inhibits miR-122-5p to elevate the expression of key glycolytic genes such as aldolase A (*ALDOA*) and *PKM*, thereby boosting glucose uptake, ATP production and glycolysis [117].

The lncRNA-driven regulation of lipid metabolism substantially contributes to HCC progression. By reprogramming the expression of lipogenic enzymes, lncRNAs enhance lipid synthesis and thereby support HCC initiation and progression [118].

Accumulating evidence shows that lncRNAs engage in multiple biological events, such as cell cycle regulation, proliferation, apoptosis and cell death, and they modulate cancer growth, invasion and metastasis [119,120]. Elevated fatty acid buildup in tumor cells accelerates HCC progression via enhanced fatty acid synthesis or impaired fatty acid oxidation [121]. As a ceRNA, lncRNA RP11-386G11.10 sequesters miR-345-3p to regulate nucleoprotein HNRNPU and its downstream lipogenic enzymes including FAS, triggering lipid deposition in HCC cells [122]. lncRNA RP11-817l4.1 promotes FA synthesis via the miR-3120-3p/ATP-citrate lyase (ACLY) axis, thereby accelerating the onset and progression of HCC [123]. lncRNA NEAT1 promotes aberrant lipolysis in HCC cells by upregulating ATGL, and the combined detection of NEAT1 and ATGL also enhances the precision of prognostic evaluation for HCC patients [124]. Wang et al. exhibited that lncRNA LINC01468 enhances HCC cell proliferation by stimulating lipogenesis. It facilitates the Cullin 4A (CUL4A)-dependent ubiquitin degradation of SHIP2, which in turn activates the PI3K/AKT/mTOR signaling pathway to induce de novo lipogenesis [125]. Two lncRNA members of the small nucleolar RNA host gene family, *SNHG1* and *SNHG7*, modulate critical effectors involved in the FA metabolism, including glycerol kinase 2 and carnitine palmitoyl-transferase 2, and thereby facilitate FA β-oxidation [126].

In addition, disrupted cholesterol metabolism serves as a key contributor to oncogenic progression. Tissue specimens collected from 30 HCC patients exhibit the marked upregulation of lncRNA LINC00618. This lncRNA accelerates tumor expansion and metastatic spread by stimulating cholesterol biosynthesis, which stabilizes NSUN2 protein and subsequently increases SREBP2 mRNA stability via an m5C-dependent regulatory mechanism [127].

### 4.2. LncRNA in Metabolic Dysfunction-Associated Steatotic Liver Disease (MASLD)

Over recent decades, the nomenclature for metabolic-origin fatty liver disease has evolved alongside updates to diagnostic frameworks. The historically dominant term NAFLD adopts an exclusion-based diagnostic criterion, requiring elimination of excessive alcohol intake and other secondary causes of hepatic steatosis. In 2020, an international expert consensus proposed MAFLD as a replacement, shifting to a positive diagnostic standard defined by hepatic steatosis plus evidence of metabolic dysfunction [4,128]. In 2023, a joint consensus from three major global liver associations updated the terminology to metabolic dysfunction-associated steatotic liver disease (MASLD) to reduce stigma and align naming with pathophysiological features, with diagnostic criteria largely consistent with MAFLD [129,130].

For narrative consistency, this review primarily uses the term MASLD throughout the main text. For individual original studies that adopted NAFLD or MAFLD diagnostic criteria, we preserve the original study definition and interpret all findings within their respective diagnostic frameworks. MASLD and its historical designation NAFLD represent one of the most prevalent etiologies of liver disorders globally, and impose a substantial and growing public health burden. A systematic review and meta-analysis of 245 imaging-based studies encompassing 5,399,254 individuals from 25 countries reported that prevalence rose from 21.9% in 1991 to 37.3% in 2019, with estimates varying by population, age, adiposity, and diagnostic modality [131,132]. Unresolved mechanistic details, the shortage of early screening tools, and lack of appropriate biomarkers may account for this limitation. But the accumulation of excess lipid within hepatocytes is the hallmark of MASLD.

lncRNA SNHG4 mitigates FFA-induced lipid accumulation and inflammation in MASLD hepatocytes by sponging miR-34b-5p to restore X-linked inhibitor of apoptosis protein (XIAP) expression. This change elevates lipid catabolism-related PPARα, carnitine palmitoyltransferase 1A (CPT1A) and ABCA1, thereby reducing hepatic lipid deposition [133]. lncRNA Gm12664-001 alleviates hepatic steatosis in AML12 cells on the bioinformatic level by repressing miR-295-5p, which in turn upregulates CAV1 and reduces lipid accumulation without any luciferase reporter assays, RNA immunoprecipitation, RNA pull-down assays, or rescue experiments [134]. Similarly, in AML12 hepatocytes, cytoplasmic lncRNA CidecAS directly associates with and stabilizes ATP1a1 to stimulate Na^+^/K^+^-ATPase activity. It activates AMPK signaling and upregulates lipolytic and fatty acid oxidation genes such as ATGL, hormone-sensitive lipase (HSL), CPT1 and ACOX1 to reduce hepatic lipid accumulation, as well as circulating triglycerides and hepatic steatosis, in mice with MASLD [135]. Meng et al. showed that lncRNA MEG3 restrains de novo lipogenesis in HepG2 hepatocytes via restraining FOXO1 nuclear shuttling and thereby lowering the abundance of downstream ACC1 and FAS [136]. Huang et al. found that MEG3 also mitigates excessive lipid deposition by binding miR-21 and modulating LRP6 expression [137]. Silencing lnc_217 not only represses de novo lipogenesis by blocking SREBP-1c cleavage, but also enhances fatty acid β-oxidation by upregulating peroxisome proliferator-activated receptor α and carnitine palmitoyltransferase-1α [138]. The downregulation of lncRNA NEAT1 alleviates MASLD progression via the mTOR/ribosomal protein S6 kinase B1 (S6K1) pathway [139]. lncRNA AC012668 overexpression suppresses lipo-related gene expression and triacylglycerol deposition by sequestering miR-380-5p away from lipoprotein-related protein LRP2 in LO2 cells exposed to free fatty acids and mice receiving a high-fat diet [140].

As a ceRNA, lncRNAs can regulate lipid metabolism by sponging kinds of microRNAs. Liver-enriched lncRNA Gm15622 exacerbates hepatic lipogenesis in MASLD by sponging miR-742-3p to upregulate lipogenic SREBP-1c level and stimulate hepatic de novo lipogenesis and lipid deposition, while metformin alleviates lipid deposition by inhibiting Gm15622 [141]. Similarly, elevated lncRNA MALAT1 acts as a ceRNA to sponge miR-690 in MASLD hepatocytes to block lipid autophagy and aggravate hepatic lipid accumulation [142]. MALAT1 sponges miR-206 and upregulates ARNT to transcriptionally repress PPARα, elevates CD36 expression, and ultimately accelerates hepatic lipid biosynthesis and accumulation to aggravate MASLD [143]. Histone H3K27 acetylation activates lncRNA NEAT1 to sequester miR-212-5p and de-repress GRIA3, which upregulates lipogenic ACC1 and FAS and accelerates hepatic lipid accumulation to drive MASLD progression [144]. By sequestering miR-139-5p to control the c-Jun/SREBP-1c cascade, lncRNA NEAT1 amplifies FFA-provoked lipid accumulation in hepatocytes, which supports its potential as a therapeutic candidate for MASLD [145]. Upregulated lncRNA PVT1 in patients and mice with MASLD targets for, and possibly sponges, miR-20a-5p to exacerbate lipid accumulation and insulin resistance under the condition of non-RIP and non-pull-down assay [146]. Liu et al. showed that lncRNA ZFAS1 also acts as a ceRNA to sequester miR-144-5p, leading to increased lipid deposition and oxidative stress in patients with MASLD and mouse models fed with HFD [147]. Further, upregulated lncRNA HOTAIR acts as a ceRNA to sequester miR-130b-3p in FFA-treated HepG2 cells and primary hepatocytes. This effect elevates ROCK1 expression, suppresses AMPK signaling and promotes lipogenic genes expression, like *FASN*, *ACACA* and *SREBP-1c*, to aggravate hepatic triglyceride and cholesterol accumulation [148]. lncRNA GAS5 aggravates hepatic lipid accumulation by sequestering miR-26a-5p, which elevates phosphodiesterase 4B (PDE4B) and represses the cAMP/CREB signaling cascade. This event enhances de novo lipogenesis and impairs mitochondrial function in HFD-fed mice and FFA-challenged hepatocytes [149]. In addition, lncRNA Gm28382 is upregulated in palmitic acid-induced AML12 cells and HFD-induced mice to sequester miR-326-3p to lift the suppression of lipogenic transcription factor ChREBP, which increases the expression of ACC1, FAS and other lipid-synthetic proteins to lipid accumulation [150]. Furthermore, lncRNA RMRP is markedly upregulated in MASLD patient livers and fatty liver rats or hepatocyte models, acting as a lipogenic driver, and sponges to sequester anti-lipogenic miR-206, thereby elevating protein tyrosine phosphatase non-receptor type 1 (PTPN1) expression. Activated PTPN1 triggers the protein phosphatase 2A (PP2A)/specificity protein 1 (SP1)/SREBP-1c cascade, upregulating lipogenic genes *FASN*, *SCD1* and glycerol-3-phosphate acyltransferase (*GPAT*) to boost triglyceride accumulation and hepatic steatosis [151]. Upregulated lncRNA HCG18 sponges miR-197-3p, upregulating IL18 to promote hepatic lipid accumulation in MASLD [152]. Acting as a ceRNA for miR-613, lncRNA CCAT1 enhances LXRα transcription in an LCRE-dependent manner, supporting lipid droplet biogenesis and accelerating MASLD progression [153]. Oppositely, lncRNA LINC01260 induces RXRB transcription and translation to significantly inhibit the steatosis process in the oleic acid-induced MASLD LO2 cells model on the bioinformatic level only, without any validation experiments [154]. lncRNA Mirt2 elevates USP10 expression to attenuate hepatic steatosis and MASLD by sponging miR-34a-5p [155]. lncRNA GAS5 sponges miR-28a-5p to relieve the miR-dependent suppression of the E3 ubiquitin ligase MARCH7. MARCH7 in turn drives the proteasomal degradation of NOD-like receptor family pyrin domain-containing 3 (NLRP3) and suppresses NLRP3-indeuced pyroptosis, alleviating HFD-induced hepatic steatosis, inflammation and insulin resistance in mice [156].

Aside from ceRNA, lncRNA AK142643 is upregulated in the liver tissues of HFD-fed mice. It enhances hepatic lipid accumulation by promoting the interaction between insulin-like growth factor 2 mRNA binding protein 2 (IGF2BP2) and CD36 mRNA, which improves CD36 mRNA stability and elevates its expression [157]. lncRNA regulator of hyperlipidemia (RHL) binds directly to hnRNPU to stabilize this protein and activate Bmal1, leading to suppressed VLDL secretion in hepatocytes, thereby resulting in lipid accumulation and the destruction of lipid metabolism [158]. Moreover, lncARSR enhances hepatic lipogenesis and contributes to hepatic steatosis in MASLD by upregulating SREBP-1c level via the PI3K/AKT pathway in MASLD patients and a fatty-acid-treated mouse model [159]. lncRNA SRA promotes hepatic steatosis primarily by inhibiting the inductive effects of the transcription factor FoxO1 to downregulate the expression of adipose triglyceride lipase (ATGL) in high-fat-diet-induced SRA-knockout mice [62]. Under the unique humanized liver mouse model, the lncRNA hLMR1 showed high expression in individuals homozygous for re9653945-G, contributing to the development of hyperlipidemia and MASLD [160]. In the liver of HFD-induced obesity mice, palmitic acid promotes Snhg3 expression, which interacts with SND1 protein to stabilize it, facilitate its nuclear location and promote K63-linked ubiquitination. This triggers SND1-dependent chromatin remodeling that lowers the H3K27me3 level at the Pparg promoter and upregulates PPARγ to increase lipid accumulation and steatosis progression [161]. In the lncRNA Gm15441 mouse knockout model, the loss of Gm15441 facilitates NLRP3 inflammasome activation alongside hepatic lipid buildup, and this phenotype occurs in the context of PPARα stimulation [162]. Similarly, acute fasting in G23Rik-deficient mice triggers hepatic lipid buildup, accompanied by elevated cholesterol and triacylglycerol levels driven by PPARα-dependent signaling [163]. lncRNA Hilnc physically interacts with IGF2BP2 to stabilize Pparg mRNA, and activates PPARγ signaling to promote lipid accumulation and hepatic steatosis [164].

### 4.3. LncRNA in Type 2 Diabetes Mellitus

Type 2 diabetes mellitus (T2DM) is a complex and multifactorial metabolic disorder. According to the IDF Diabetes Atlas (11th edition, 2025), an estimated 588.7 million adults aged 20–79 years (11.1%) were living with diabetes worldwide in 2024, and this number is projected to reach 852.5 million by 2050 [165]. As a major global health challenge, diabetes mellitus affects millions of people on the global level. It interferes with the normal glucose metabolism, insulin utilization and blood sugar level, and it contributes to a series of health conditions, including cardiovascular diseases, tumors, chronic metabolic diseases, retinal lesions and so on. Diabetes also places substantial health and economic burdens on numerous individuals, families and healthcare systems [166]. While this review centers on hepatic metabolic regulation, lncRNAs in pancreatic β-cells and peripheral tissues are included for their direct upstream effects on liver function. Altered insulin secretion and peripheral fatty acid flux directly shape hepatic insulin signaling, lipogenesis and gluconeogenesis, forming an integral part of the hepatic regulatory network.

T2DM and its complications represent severe threats to public health, and early preventive strategies targeted at high-risk populations can mitigate these adverse outcomes for high-risk groups. Nevertheless, conventional risk models exhibit limited performance in forecasting personal diabetes likelihood [167]. Nowadays, numerous lncRNAs participating in the pathogenic mechanisms of T2DM are promising diagnostic biomarkers and therapeutic targets for type 2 diabetes mellitus [168]. As a protective lncRNA, GAS5 improves pancreatic β-cell insulin secretion and peripheral insulin sensitivity by sponging multiple miRNAs to activate insulin receptor (INSR)/PI3K/AKT signaling, which inhibits renal fibrosis and diabetic complications, and serves as a serum biomarker for type 2 diabetes [169]. Similarly, the analysis of serum samples collected from US military veterans demonstrated that the combined measurement of circulating GAS5 and other biochemical markers improves diagnostic performance for diabetes [170]. In a case-control study, upregulated circulating lncRNA LINC01018 shows favorable discriminative performance for distinguishing T2DM patients from healthy controls, supporting its potential as a diagnostic biomarker. Functional experiments further demonstrated that LINC01018 restrains miR-499a-5p and exacerbates high-glucose-induced pancreatic β-cell dysfunction [171]. Its ability to predict future T2DM incidence remains to be validated in prospective population cohorts. Serum elevated lncRNA LINC-P21 is a potential discriminative tool between T2DM patients and healthy persons, serving as a potential regulator in insulin secretion and the proliferation of pancreatic β cells by targeting miR-766-3p to upregulate NR3C2 without lncRNA–miRNA binding validation [172]. Elevated circulating lncRNA-CR848007.6 acts as a potential independent risk factor for type 2 diabetes mellitus (T2DM). This transcript may function as a promising biomarker reflecting cadmium-induced injury in pancreatic β-cells and T2DM pathogenesis [173]. Through the transcriptome analysis of abnormally expressed lncRNAs in patients with T2DM, Ma et al. found that lncRNA XR_108954.2 is a potential biomarker for T2DM [174]. Through a case-control study, Omidvar et al. found downregulated expression levels of lncRNA VIM-AS1 and CTBP1-AS2 in T2DM patients, with low CTBP1-AS2 expression serving as a potential independent risk factor or showing moderate discriminative diagnostic performance for T2DM [175]. Additionally, peripheral blood mononuclear cells (PBMCs)-derived lncRNA LY86-AS1 and HCG27_201 were significantly downregulated in T2DM patients and negatively correlated with fasting blood glucose, with LY86-AS1 potentially showing moderate diagnostic efficacy for T2DM [176]. Xu et al. reported that the combined detection of lncRNA SNHG1 and miR-195 achieves good discriminative accuracy for T2DM in a case-control setting. Mechanistically, SNHG1 sequesters miR-195 to curb proliferation and insulin secretion while triggering apoptosis in INS-1 pancreatic β-cells [177]. Evidence from prospective cohorts is still required to support its predictive value for incident T2DM. Similarly, the deregulation of lncRNA SNHG17 and TTC28-AS1 is associated with T2DM [178]. The upregulation of lncRNA MEG3 plays as an important role in diabetic-related vascular complications’ progression, likely serving as potential diagnostic marker for T2DM [179]. Additionally, the rs10757279 polymorphism in antisense non-coding RNA in the INK4 locus (ANRIL) gene is strongly associated with genetic susceptibility to T2DM [180]. Notably, most current biomarker evidence is derived from case-control studies, which support the discriminative diagnostic capacity of lncRNAs for prevalent T2DM. Large-scale prospective cohort studies are still required to validate whether these circulating lncRNAs can predict the risk of future T2DM onset in at-risk populations.

H19 downregulation impairs ER–mitochondria coupling and insulin signaling to augment hepatic gluconeogenesis in diabetic hyperglycemia [78]. The rs3819316 C > T mutation of lncRNA Reg1cp elevates T2DM risk by binding polypyrimidine tract binding protein 1 (PTBP1) to damage pancreatic β-cell insulin secretion and spreading through islet exosomes to suppress peripheral AdipoR1 expression and induce systemic insulin resistance [181]. Wang et al. found that BMAL1-regulated lncCplx2 recruits EZH2 to silence circadian genes and maintain normal β-cell insulin secretion and glucose homeostasis, whereas its deficiency causes glucose intolerance and diabetic hyperglycemia [182]. Overexpressed LINC-p21 functions as a ceRNA to sequester miR-335-3p, which increases insulin-like growth factor 1 (IGF-1) abundance. This cascade induces pancreatic β-cell apoptosis, defective insulin release, and glucose intolerance in T2DM both in vitro and in vivo [183]. In children and adolescents, the lncOb rs10487505 variant modulates circulating leptin concentrations and exacerbates insulin resistance, raising prediabetes susceptibility in obese youth [184]. In addition, Liao et al. found that upregulated lncRNA EPB41L4A-AS1 in the livers of patients with T2DM suppresses glucose uptake by targeting glucose transporter (GLUT) 4/2 and TXNIP. It interacts with GCN5 to regulate histone and nonhistone acetylation or crotonylation, thereby aggravating T2DM progression [185].

By activating the PI3K/AKT/mTOR cascade, lncRNA p3134 upregulates critical regulatory factors and amplifies glucose-stimulated insulin release in pancreatic β-cells. It restores insulin synthesis and secretion in db/db mice to preserve β cell function for T2DM [186]. lncRNA SOX2OT ameliorates insulin resistance via IRS/PI3K/AKT signaling activation and the miR-552-5p/SOX2-mediated repression of inflammation and oxidative stress in T2DM [187]. Kong et al. found that the overexpression of lncRNA LEGLTBC regulates the miR-34a/sirtuin 1 (SIRT1) axis to relieve glucotoxicity-induced oxidative stress and apoptosis [188]. Besides this, lncRNA KDM4A-AS1 directly targets to miR-423-5p to modulate the expression of downstream growth differentiation factor 11, which exhibits a protective effect on pancreatic β cells, leading to increased glucose-induced insulin secretion and decreased cell apoptosis rate [189]. lncRNA TUG1 suppresses miR-204 expression and stimulates the SIRT1/AMPK/ACC axis, which confers protective effects and restrains diabetic pathogenesis [190]. Moreover, lncRNA uc.322 is enriched in pancreatic β-cells. It elevates the levels of insulin transcriptional regulators pancreatic and duodenal homeobox 1 (PDX1) and Forkhead box O1 (FOXO1), enhancing insulin secretion to exert anti-diabetic effects [191]. lncRNA ROIT protects against T2DM by destabilizing DNMT3A to maintain Nkx6.1-driven insulin production and glucose-stimulated insulin secretion [192]. Interestingly, resveratrol and metformin alleviate high-fat-diet-induced hepatic insulin resistance in mice by altering hepatic lncRNA profiles. NONMMUT034936.2 regulates the PI3K/AKT pathway to suppress gluconeogenetic genes for the treatment of insulin resistance and diabetes [193]. Exercise ameliorates insulin resistance by inhibiting lncRNA MALAT1, which elevates miR-382-3p expression and suppress resistin for T2DM therapy [194].

To sum up, insulin resistance is a key step of T2DM and various metabolic diseases or syndromes. From glycolysis to gluconeogenesis, any dysregulation of these processes will drive the onset of diabetes mellitus phenotypes. Exploring and studying these lncRNAs has revealed to us an association between lncRNAs and the development of T2DM, revealing the mechanisms and action targets of anti-diabetic treatment, such as resveratrol and metformin [195]. Meanwhile, lncRNA provides us different insights into early-stage diagnostics for T2DM.

### 4.4. LncRNA in Gestational Diabetes Mellitus

Gestational diabetes mellitus (GDM) represents a complicated metabolic disorder typically identified between 24 and 28 weeks of pregnancy [196]. Reported prevalence varies substantially with population and diagnostic criteria, such as the IADPSG/WHO one-step and two-step Carpenter–Coustan protocols. Under the 2024 IDF estimates, GDM accounts for approximately 15–16% of live births globally and varies by region [197]. GDM manifests as impaired glucose tolerance and hyperglycemia, and raises the likelihood of multiple gestational complications such as preeclampsia, stillbirth, and metabolic abnormalities in newborns, as well as increasing the risk of developing T2DM and other metabolic diseases for women with GDM [198]. Growing evidence has increasingly highlighted the contributions and modulations of lncRNA to GDM progression, such as regulating transcription, epigenetic remodeling and post-transcriptional control in hepatic gluconeogenesis and insulin resistance [199,200].

Promoter hypomethylation drives the epigenetic upregulation of lncRNA MEG3 in the livers of F1 offspring of GDM mice, resulting in elevated hepatic gluconeogenesis, and impaired insulin sensitivity and glucose tolerance [201]. In addition, placenta-derived extracellular vesicle lncRNAs in GDM act as interorgan signaling messengers to disrupt hepatic PI3K/AKT-FoXO1 signaling, enhance excessive hepatic gluconeogenesis and aggravate maternal hepatic insulin resistance, and further induce the persistent epigenetic reprogramming of fetal liver glucose metabolism, thereby raising offspring’s long-term diabetes risk [202].

## 5. Discussions and Future Prospects

LncRNAs exert pleiotropic effects on glucose and lipid metabolism in both physiological and pathological contexts, which participate in the modulation of diverse biological processes in various tissues and are implicated in multiple diseases, especially in hepatic metabolic-related diseases, including MASLD, HCC, and T2DM. Although lncRNAs have been regarded as non-functional transcripts, the lncRNA gene can be transcribed and spliced into mature lncRNA that mediates specific effects in different cellular modulatory pathways. Furthermore, lncRNAs exert distinct functions depending on their subcellular localization in the nucleus or the cytoplasm. Through transcriptional, post-transcriptional, and epigenetic mechanisms, lncRNAs do not regulate cholesterol, triglyceride, and glucose metabolism independently. Rather, they coordinate these pathways centered on the PI3K/AKT, AMPK, SREBP, PPAR, and FoxO1 signaling networks. The PI3K/AKT/mTORC1 axis couples glucose disposal with lipid storage, as AKT suppresses FoxO1-driven gluconeogenesis while mTORC1 promotes SREBP-1c-dependent lipogenesis. AMPK acts in the opposite direction to suppress lipogenesis, promote fatty acid oxidation, and restrain gluconeogenesis. At the nuclear level, SREBPs, PPARs, and FoxO1 jointly coordinate cholesterol, triglyceride, and glucose outputs. At the gene expression level, pleiotropic lncRNAs such as H19, MEG3, MALAT1, and lncARSR simultaneously influence lipogenesis, fatty acid oxidation, insulin signaling, and gluconeogenesis rather than acting on a single compartment. At the metabolic functional level, this coordinated control links hepatic steatosis to insulin resistance and glucose imbalance. In this way, lncRNAs form an upper regulatory layer that integrates lipid and glucose signals into a coordinated hepatic metabolic network. Beyond the liver, lncRNAs in adipose tissue, macrophages, and pancreatic β-cells also shape hepatic metabolism through circulating free fatty acids, reverse cholesterol transport, and insulin input, thereby extending this network across multiple tissues. Characterizing this network as a whole, rather than pathway by pathway, will help clarify how its dysregulation contributes to MASLD, HCC, and diabetes [203,204].

Tissue expression profiles of lncRNAs correlate with different stages of varied diseases. As such, lncRNAs are increasingly considered candidate molecules for further preclinical investigation in disorders of glucose and lipid homeostasis. Recently, more studies have also examined the potential of LncRNAs as diagnostic or prognostic biomarkers in tumors and T2DM, amongst others. Moreover, the distinct effects of lncRNA in diverse tissues and pathological states unfold the complexity of lncRNA functions in vivo. The present review seeks to improve the current understanding of lncRNAs in the glucose and lipid metabolism of liver and hepatic-related diseases, which may provide novel signaling targets for developing potential therapeutic interventions into many metabolic diseases.

Current studies of these lncRNAs remain at an early stage, and several critical limitations should be acknowledged in the present review. Firstly, the precise molecular mechanisms of lncRNA interaction in different biological processes are not clear. The most extensively studied is still the role of lncRNA in tumors, but research on hepatic energy metabolism is not comprehensive. In addition, the difficulty of measuring lncRNA in the circulation or tissue is another limit for developing lncRNA as a potential therapeutic or diagnostic tool for treating diseases. Further, it is difficult to translate research from animals to humans due to the low-conserved DNA sequence of lncRNAs in different species. Therefore, future work on the mechanisms and interactions of lncRNAs in hepatic glucose and lipid metabolism is needed to resolve the above issues.

Additionally, it is noteworthy that lncRNA H19 and GAS5 have been reported to exert opposing effects on the same hepatic endpoint, and these apparent discrepancies mainly reflect differences in experimental context rather than true contradictions.

For H19 in gluconeogenesis, Zhang et al. showed that hepatic H19 overexpression in diabetic mice promotes gluconeogenesis through Hnf4a-mediated transcriptional control, whereas Nandwani et al. and others reported that H19 knockdown aggravates gluconeogenesis through impaired insulin signaling and FoxO1 activation [78,79]. These two lines differ in nutritional or disease context (e.g., diabetic liver in which H19 is already induced versus basal hepatocytes), direction of intervention (e.g., overexpression versus loss-of-function), and subcellular level (e.g., nuclear epigenetic control of Hnf4a versus cytoplasmic or mitochondrial control of insulin signaling and FoxO1 nuclear entry). Likewise, GAS5 aggravates cholesterol and triglyceride accumulation in some settings, yet protects against hepatic inflammation and preserves pancreatic β-cell function in others. These discrepancies track with the distinct miRNA partners that GAS5 engages in hepatocytes, macrophages, and β-cells, and with the endpoint measured, whether cholesterol synthesis, triglyceride lipogenesis, inflammatory cell death, or glycogen storage. At present, the available evidence does not fully resolve these context-dependent roles, and matched head-to-head comparisons are required before H19 or GAS5 can be assigned a uniform pro- or anti-metabolic function. This context dependency represents a major challenge for translating lncRNA findings into universal and potential diagnostic or therapeutic strategies.

For clinical translation, key problems remain alongside these mechanistic gaps. Standardized and validated protocols for lncRNA detection in clinical samples are lacking, including liver tissue and circulating specimens, leading to the poor consistency of biomarker performance across independent cohorts. Safe and efficient hepatocyte-targeted delivery systems of lncRNA-based therapeutics, like exogenous viral vector and endogenous exosomes, with minimal off-target effects and low immunogenicity, also represent a major technical bottleneck [205]. Future research should prioritize human-specific lncRNAs with well-validated causal roles, integrate multi-omic data to map lncRNA-centered regulatory networks, and develop liver-targeted nucleic acid delivery platforms to bridge the gap between basic discoveries and clinical application.

As for evolutionary conservation and cross-species translational limitations, unlike protein-coding genes, most lncRNAs exhibit low sequence conservation across species and frequently show species-specific expression patterns and molecular interaction profiles. Several lncRNAs including H19, MEG3, MALAT1 and GAS5 have confirmed human orthologs, with conserved hepatic metabolic roles validated in both human hepatocytes and rodent models. In contrast, the majority of functionally identified lncRNAs, such as most murine Gm-series transcripts and several rat-specific loci, have no verified human orthologs, and their metabolic regulatory effects have only been demonstrated in rodent experimental systems. This discrepancy means preclinical findings from animal models cannot be directly translated to human clinical applications.

## 6. Conclusions

Despite these limitations, lncRNAs have been shown to contribute to the regulation of hepatic metabolic disorders in preclinical models, which suggests their potential as candidate biomarkers and intervention targets for further investigation. Current evidence remains largely experimental, and whether these findings can be translated into clinical diagnosis or treatment requires validation in human cohorts.

## Figures and Tables

**Table 1:** LncRNAs regulating hepatic cholesterol metabolism.

LncRNA	Experimental Model (In Vitro/In Vivo/Human)	Molecular Target	Mechanism and Regulatory Direction	Molecular Validation Approach	Affected Metabolic Process	Evidence Level	Ref.
**Cholesterol biosynthesis**							
LncARSR	Human: hypercholesterolemic patient specimens In vivo: C57BL/6J HCD mice In vitro: HepG2 hepatocyte	PI3K/Akt SREBP-2 HMGCR	Activates PI3K/Akt, upregulating SREBP-2, and drives HMGCR to promote de novo cholesterol synthesis	ChIP-qPCR promoter reporter/luciferase qPCR/WB of target gene	De novo cholesterol biosynthesis	A	[21,22]
LeXis	In vivo: C57BL/6J mice In vitro: GW3695-treated primary mouse hepatocytes	RALY SREBP-2 promoter	Binds RALY and inhibits RALY occupancy at the SREBP-2 promoter to reduce hepatic/serum cholesterol	ChIP-qPCR promoter reporter/luciferase qPCR/WB of target gene	Cholesterol biosynthesis	B	[23,24]
AT102202	In vitro: cultured human HepG2 hepatocytes	HMGCR	Negatively regulates HMGCR expression to lower cholesterol biosynthesis	ChIP-qPCR promoter reporter/luciferase qPCR/WB of target gene	Cholesterol biosynthesis	C	[25]
GAS5	In vitro: FFA-induced human HepG2 hepatocytes In vivo: HCD mouse liver	miR-29a-3p/NOTCH2 HMGCR	Acts via miR-29a-3p/NOTCH2, relieving miRNA-mediated suppression of HMGCR	Dual-luciferase reporter assay lncRNA/miRNA qPCR target mRNA and protein (WB) RIP/RNA pull-down	Cholesterol synthesis hepatic accumulation	B	[26]
HOXC-AS1	In vitro: THP-1 macrophages	HOXC6	Upregulates HOXC6, overexpression suppresses Ox-LDL-induced intracellular cholesterol accumulation.	ChIP-qPCR promoter reporter/luciferase qPCR/WB of target gene	Ox-LDL cholesterol accumulation	E	[27]
TRIBAL	Human: liver tissue cohort In vitro: Primary human hepatocytes	Cholesterol biosynthesis and esterification gene set	Regulates hepatic cholesterol handling and synthesis, knockdown downregulates biosynthesis and esterification genes, efflux unchanged	ChIP-Qpcr promoter reporter/luciferase qPCR/WB of target gene	Cholesterol biosynthesis and esterification	B	[28]
**Cholesterol transport and efflux**							
lincRNA-DYNLRB2-2	In vitro: THP-1 macrophages In vivo: apoE^−/−^ mice	GPR119 GLP-1R ABCA1	upregulates GPR119/GLP-1R signaling to enhance ABCA1 efflux. Peripheral efflux feeds reverse cholesterol transport to the liver.	ChIP-qPCR promoter reporter/luciferase qPCR/WB of target gene	ABCA1-mediated cholesterol efflux	**E**	[29]
MeXis	In vitro: Primary mouse thioglycollate-elicited peritoneal macrophages In vivo: MeXis knockout C57BL/6 mouse	DDX17 Abca1 locus	Binds DDX17 to enhance Abca1 chromatin accessibility, promoting efflux and suppressing lesions	ChIP-qPCR promoter reporter/luciferase qPCR/WB of target gene	ABCA1 efflux atherosclerosis	E	[30]
DAPK1-IT1	In vitro: human THP-1 macrophages In vivo: apoE^−/−^ mice with HFD	hsa-miR-590-3p LPL	Forms the miR-590-3p/LPL axis, elevating LPL, impairing efflux and boosting lipid uptake	Dual-luciferase reporter assay lncRNA/miRNA qPCR target mRNA and protein (WB) RIP/RNA pull-down	Efflux impaired lipid uptake	E	[31]
RP5-833A20.1	In vitro: THP-1 macrophages In vivo: apoE^−/−^ mice	miR-382-5p NFIA	Upregulates miR-382-5p and downregulates NFIA and diminishes macrophage efflux.	Dual-luciferase reporter assay lncRNA/miRNA qPCR target mRNA and protein (WB) RIP/RNA pull-down	Macrophage efflux	E	[32]
Lnc-HC	In vitro: primary rat hepatocytes In vivo: HCD-induced metabolic syndrome rats	C/EBPβ hnRNPA2B1 Cyp7a1, ABCA1 mRNA	hnRNPA2B1 complex binds and destabilizes Cyp7a1 and ABCA1 mRNAs	RIP actinomycin-D mRNA stability chase target mRNA/protein WB	Bile-acid synthesis and ABCA1 efflux	B	[33]
MALAT1	In vitro/in vivo: macrophage atherosclerosis model	NF-κB ABCA1, ABCG1	Activates NF-κB and reduces ABCA1/ABCG1 and suppressing macrophage efflux	ChIP-qPCR promoter reporter/luciferase qPCR/WB of target gene	Macrophage efflux	E	[34]
ZFAS1	In vitro: ox-LDL-induced macrophages	miR-654-3p ADAM10 RAB22A	ceRNA sponge of miR-654-3p, elevating ADAM10/RAB22A	Dual-luciferase reporter assa lncRNA/miRNA qPCR target mRNA and protein (WB) RIP/RNA pull-down	Macrophage efflux	E	[35]
CDKN2B-AS1	In vitro: THP-1 macrophages In vivo: male apoE^−/−^ SPF mice	DNMT1 ADAM10 promoter	Binds DNMT1 to methylate the ADAM10 promoter, suppressing ADAM10 and inflammation	ChIP-qPCR (histone marks) promoter methylation assay qPCR/WB	Macrophage efflux	E	[36]
CHROME	In vitro: HepG2 human hepatocarcinoma cells	miR-27b, miR-33a/b, miR-128 ABCA1	Sequesters these miRNAs and derepresses ABCA1 under LXR/high-cholesterol conditions	Dual-luciferase reporter assay lncRNA/miRNA qPCR target mRNA and protein (WB) RIP/RNA pull-down	Cholesterol efflux HDL biogenesis	B	[37,38]
PCA3	In vitro: ox-LDL-induced macrophages In vivo: male apoE^−/−^ mice	miR-140-5p RFX7 ABCA1	Sponges miR-140-5p to upregulate RFX7 and activates ABCA1, enhancing efflux and RCT	Dual-luciferase reporter assay lncRNA/miRNA qPCR target mRNA and protein (WB) RIP/RNA pull-down	Macrophage efflux	E	[39]
lincRNA DYNLRB2-2	In vitro: THP-1 and mouse RAW264.7 macrophages	TLR2 ABCA1	Downregulates TLR2 to increase ABCA1, enhancing efflux and reducing intracellular accumulation/foam cells	ChIP-qPCR promoter reporter/luciferase qPCR/WB of target gene	Macrophage efflux	E	[40]
NIPA1-SO	In vitro: THP-1 macrophages In vivo: atherosclerotic animal models	BMPR2-Smad1/5/8 ABCA1, ABCG1	Enhances ABCA1/ABCG1 via BMPR2-Smad1/5/8 to reduce intracellular load and improves lipids	ChIP-qPCR promoter reporter/luciferase qPCR/WB of target gene	Efflux circulating lipid profile	E	[41]
Hnf1aos1	In vitro: Hepa-1c1c7 mouse hepatoma cell line In vivo: C57BL/6J male mice	CYP7A1 ABCA1 APOB	Knockdown depletes CYP7A1 and disrupts breakdown, compensatory upregulation of ABCA1/APOB enhances hepatocyte efflux	ChIP-qPCR promoter reporter/luciferase qPCR/WB of target gene	Cholesterol breakdown and efflux	B	[42]
DANCR	In vitro: THP-1 and mouse RAW264.7 macrophages	ABCA1, ABCG1, SR-BI CD36, SR-A	Downregulates efflux transporters ABCA1/ABCG1/SR-BI and upregulates uptake receptors CD36/SR-A	ChIP-qPCR promoter reporter/luciferase qPCR/WB of target gene	Efflux suppressed/ uptake enhanced	E	[43]
MEG3	In vitro: ox-LDL-induced human endothelial cells	DNMT3B miR-181a-5p ABCA1	DNMT3B-mediated MEG3 downregulation acts via miR-181a-5p/ABCA1 to suppress efflux	Dual-luciferase reporter assay lncRNA/miRNA qPCR target mRNA and protein (WB) RIP/RNA pull-down	ABCA1 efflux	E	[44]
KCNQ1OT1	In vitro: THP-1 macrophages	miR-452-3p HDAC3 ABCA1	Competitively binds miR-452-3p to upregulate HDAC3 and downregulates ABCA1	Dual-luciferase reporter assa lncRNA/miRNA qPC target mRNA and protein (WB) RIP/RNA pull-down	Macrophage efflux	E	[45]
APAT	In vitro: THP-1 macrophages In vivo: C57BL/6N mice	CD36 SR-B1 ABCG1	Upregulates CD36 and represses SR-B1/ABCG1, promoting accumulation and suppressing efflux in vitro and in vivo	ChIP-qPCR promoter reporter/luciferase qPCR/WB of target gene	Macrophage efflux	E	[46]
GAS5	In vivo: ApoE^−/−^ mice	EZH2 ABCA1 (histone methylation)	Knockdown reduces EZH2-mediated ABCA1 repression via histone methylation, promoting RCT and inhibiting accumulation	ChIP-qPCR (histone marks) promoter methylation assay qPCR/WB	Reverse cholesterol transport	E	[47]
APOA1-AS	In vitro: human HepG2 hepatocytes In vivo: mouse liver tissue	LSD1 PRC2 APOA1, APOC3, APOA4	Recruits LSD1/PRC2 for repressive histone marks, downregulating APOA1 and cluster genes, impairing RCT; inhibition elevates apolipoproteins and HDL-mediated efflux	ChIP-qPCR (histone marks) promoter methylation assay qPCR/WB	HDL/apolipoprotein metabolism RCT	B	[48]
H19	In vitro: human HepG2 hepatocytes In vivo: murine cholestatic fibrosis model	ZEB1 EpCAM promoter (bile-acid pathway)	Binds ZEB1 to block its EpCAM-promoter occupancy, activating EpCAM and disrupting bile-acid transport	ChIP-qPCR promoter reporter/luciferase qPCR/WB of target gene	Bile-acid transport cholesterol clearance	B	[49]
	In vitro: Huh7 and HepG2 cell lines	Bcl2 caspase-8 SHP	Bcl2-mediated caspase-8 SHP degradation derepresses H19, indirectly perturbing cholesterol transport	RNA pull-down/RIP RBP co-IP target mRNA/protein WB	Bile-acid synthesis/transport	B	[50]
MEG3	In vitro: Human Huh7 hepatocytes In vivo: cholestatic mouse model	PTBP1 Shp mRNA Cyp7a1 Cyp8b1	Scaffold recruiting PTBP1 to Shp mRNA, promoting decay and relieving Shp repression of Cyp7a1/Cyp8b1	RNA pull-down/RIP RBP co-IP target mRNA/protein WB	Cholesterol to bile acid conversion	B	[51]
APOA4-AS	In vitro: Primary hepatocytes isolated from male mice In vivo: ob/ob male mice	HuR APOA4 mRNA	Interacts with stabilizing HuR to maintain APOA4, knockdown lowers hepatic APOA4 and plasma TC	RIP actinomycin-D mRNA stability chase target mRNA/protein WB	Systemic apolipoprotein/lipid homeostasis	B	[52]
BM450697	In vitro: HepG2 and Hep3B human hepatocellular carcinoma lines	LDLR promoter	Antisense to the LDLR promoter; DNA:RNA hybrid/transcriptional interference represses LDLR, impairing LDL uptake	ChIRP/DNA:RNA hybrid assay promoter reporter LDLR qPCR and WB	Hepatic LDL uptake	C	[53]
RP1-13D10.2	In vitro: human Huh7 and HepG2 cells	LDLR transcription statin response	Overexpression promotes LDL-C clearance via LDLR transcription and modulates statin efficacy	ChIP-qPCR promoter reporter/luciferase qPCR/WB of target gene	Hepatic LDL clearance statin response	C	[54]

For evidence level, A represents that human clinical specimens/cohorts, human/mouse hepatic cell lines, in vivo animal intervention and gain/loss-of-function causal validation were used in the article. B represents that hepatic cell lines, in vivo animal models (or human tissue and cells) and causal mechanistic validation in a hepatic system were used in the article. C represents that a single-cell-line system, in silico/correlative, or overexpression/ceRNA-only without in vivo validation was utilized. E represents that the extrahepatic model was used.

**Table 2:** LncRNAs regulating hepatic glucose metabolism (glycogen, gluconeogenesis, glycolysis).

LncRNA	Experimental Model (In Vitro/In Vivo/Human)	Mechanism and Regulatory Direction	Molecular Validation Approach	Affected Metabolic Process	Evidence Level	Ref.
**Glycogen metabolism**						
LncLGR	In vitro: mouse primary hepatocytes In vivo: C57BL/6J mice liver	Recruits hnRNPL to the GCK promoter and reduces G6P and restrains glycogen synthesis	RNA pull-down/RIP RBP co-IP target mRNA/protein WB	Hepatic glycogen synthesis	B	[74]
NONRATT021972	In vivo: T2DM SD rat liver In vitro: BRL rat hepatocytes	Impairs AKT activation and represses glucokinase, silencing raises p-AKT, suppresses active GSK-3β, restores glucokinase and glycogen formation	ChIP-qPCR promoter reporter/luciferase qPCR/WB of target gene	Glycogen synthesis glucose phosphorylation	B	[75]
Snhg3	In vitro: mouse primary hepatocytes In vivo: C57BL/6J mice liver	Chromatin remodeling and elevated AKT phosphorylation upregulate PPP1R3B, boosting glycogen synthesis, improving glucose tolerance	ChIP-qPCR (histone marks) promoter methylation assay qPCR/WB	Hepatic glycogenesis glucose homeostasis	B	[76]
GAS5	In vivo: DIO diabetic mouse liver In vitro: hepatocyte models	Markedly raises Gys2, accelerating glycogen synthesis and hepatic glucose storage	ChIP-qPCR promoter reporter/luciferase qPCR/WB of target gene	Hepatic glycogen synthesis	B	[77]
**Gluconeogenesis**						
H19	In vitro: Hepa 1-6 cell lines In vivo: male C57BLKs-db + mice	H19 inhibition elevates VDAC1, activating JNK and IRS1 phosphorylation and inhibiting insulin-AKT	RNA pull-down/RIP RBP co-IP target mRNA/protein WB	Hepatic gluconeogenesis insulin signaling	B	[78]
	In vitro: HepG2 hepatocytes In vivo: db/db mice	Inhibits SAHH, reducing Hnf4a promoter methylation and drives PCK1/G6PC, boosting glucose production and hyperglycemia	ChIP-qPCR (histone marks) promoter methylation assay qPCR/WB	Hepatic gluconeogenesis	B	[79]
MEG3	In vitro: human HepG2 In vivo: diabetic rat liver	Elevates FoxO1, boosting G6PC/PEPCK, enhancing gluconeogenesis while inhibiting insulin-triggered glycogen synthesis	ChIP-qPCR promoter reporter/luciferase qPCR/WB of target gene	Gluconeogenesis; glycogen synthesis	B	[80]
	In vitro: mouse primary hepatocytes In vivo: db/db mice liver	CREB-induced ceRNA sponge of miR-302a-3p, elevating CRTC2 and activating PGC-1α-PEPCK/G6PC	Dual-luciferase reporter assay lncRNA/miRNA qPCR target mRNA and protein (WB) RIP/RNA pull-down	Hepatic gluconeogenesis	B	[81]
	In vitro: mouse primary hepatocytes In vivo: diabetic mouse liver	ceRNA sponge of miR-214, elevating ATF4-FoxO1 to boost PEPCK/G6PC, accelerating gluconeogenesis	Dual-luciferase reporter assay lncRNA/miRNA qPCR target mRNA and protein (WB) RIP/RNA pull-down	Hepatic gluconeogenesis	B	[82]
Gm10768	In vitro: mouse primary hepatocytes In vivo: db/db mice liver	Sequesters miR-214 to upregulate ATF4 and enhance gluconeogenesis	Dual-luciferase reporter assay lncRNA/miRNA qPCR target mRNA and protein (WB) RIP/RNA pull-down	Hepatic gluconeogenesis	B	[83]
Gm19619	In vitro: murine AML12 cell line In vivo: C57BL/6J mouse liver tissue	Upregulates FoxO1, elevating G6PC/Pck1, promoting gluconeogenesis and impairing insulin sensitivity	RNA pull-down/RIP RBP co-IP target mRNA/protein WB	Gluconeogenesis insulin sensitivity	B	[84]
Gomafu	In vitro: HepG2 hepatocytes In vivo: diabetic mouse liver	Sponges miR-139, abolishing FoxO1 repression, raising PEPCK/G6PC and enhancing gluconeogenesis	Dual-luciferase reporter assay lncRNA/miRNA qPCR target mRNA and protein (WB) RIP/RNA pull-down	Hepatic gluconeogenesis	B	[85]
Gm10804	In vitro: high-glucose hepatocytes In vivo: diabetic-MAFLD liver	Elevates PEPCK/G6Pase to drive glucose production, hepatic knockdown restrains gluconeogenesis and alleviates steatosis in vivo	ChIP-qPCR promoter reporter/luciferase qPCR/WB of target gene	Gluconeogenesis steatosis	B	[59]
LincIRS2	In vitro: primary mouse hepatocytes In vivo: HFD C57BL/6N obese mouse	Obesity triggers MAFG overexpression repressing LincIRS2	ChIP-qPCR promoter reporter/luciferase qPCR/WB of target gene	Gluconeogenesis insulin resistance	B	[86]
Falcor/LL35	In vitro: murine AML12 hepatocytes	Knockdown upregulates G6PC/PEPCK in AML12, LL35 negatively regulates gluconeogenesis	ChIP-qPCR promoter reporter/luciferase qPCR/WB of target gene	Gluconeogenesis	C	[87]
H19	In vivo: db/db and db/+ mouse model	Restrains gluconeogenesis by limiting p53 binding to the FoxO1 promoter, lowering FoxO1 and gluconeogenic enzymes	ChIP-qPCR promoter reporter/luciferase qPCR/WB of target gene	Hepatic gluconeogenesis	B	[88]
	In vitro: HepG2 and parimary mouse hepatocytes	Preserves insulin-AKT signaling, restricting FoxO1 nuclear entry to inhibit gluconeogenic enzymes and glucose synthesis	ChIP-qPCR promoter reporter/luciferase qPCR/WB of target gene	Gluconeogenesis insulin signaling	C	[89]
LncSHGL	In vivo: obese C57BL/6J mouse liver	Recruits hnRNPA1 to raise CALM translation, activating PI3K/AKT and inhibiting mTOR/SREBP-1c	RNA pull-down/RIP RBP co-IP target mRNA/protein WB	Hepatic gluconeogenesis	B	[90]
Bhmt-AS	In vitro: primary hepatocytes In vivo: male C57BL/6J liver model	Deficiency suppresses gluconeogenesis via Bhmt modulation and ameliorates glucose production and gluconeogenic genes in vivo	ChIP-qPCR promoter reporter/luciferase qPCR/WB of target gene	Hepatic gluconeogenesis	B	[91]
MALAT1	In vitro: HepG2, FLC4 and PHM-1 cells	Increases TCF7L2, which occupies G6PC/PCK1 promoters to repress gluconeogenic enzymes	ChIP-qPCR promoter reporter/luciferase qPCR/WB of target gene	Hepatic gluconeogenesis	C	[92]
NONMMUT031874.2	In vivo: HFD mouse model	Metformin downregulates the lncRNA, enhancing miR-7054-5p and decreasing Socs3, inhibiting PEPCK and gluconeogenesis	Dual-luciferase reporter assay lncRNA/miRNA qPCR target mRNA and protein (WB) RIP/RNA pull-down	Gluconeogenesis metformin action	B	[93]
**Glycolysis**						
H19	In vitro: Hepa1-6 and HepG2 hepatocytes In vivo: db/db and HFD C57BL/6J mouse models	Downregulation elevates DNMT3B, hypermethylating and silencing Hk2/Eno3, suppressing enzyme activity, flux and ATP production	ChIP-qPCR (histone marks) promoter methylation assay qPCR/WB	Hepatic glycolysis	B	[94]
Tug1	In vitro: HepG2 hepatocytes	ceRNA inhibiting miR-1934-3p, suppressing excessive glycolysis and reducing lipid deposition	Dual-luciferase reporter assay lncRNA/miRNA qPCR target mRNA and protein (WB) RIP/RNA pull-down	Hepatic glycolysis	C	[95]
LncLGR	In vitro/in vivo: hepatic (liver-specific) model	Binds hnRNPL to inhibit glucokinase, suppressing glycolysis and glycogen storage	RNA pull-down/RIP RBP co-IP target mRNA/protein WB	Glycolysis (suppressed); glycogen storage	B	[74]

For evidence level, B represents that hepatic cell lines, in vivo animal models (or human tissue and cells) and causal mechanistic validation in a hepatic system were used in the article. C represents that a single-cell-line system, in silico/correlative, or overexpression/ceRNA-only without in vivo validation was utilized.

## Data Availability

No new data were created or analyzed in this study.

## Abbreviations

Thefollowing abbreviations are used in this manuscript:

3PG	3-Phosphoglycerate
ABCA1	ATP-binding cassette transporter A1
ABCG1	ATP-binding cassette transporter G1
ACC1/ACACA	Acetyl-CoA carboxylase 1 (gene ACACA)
ACLY	ATP-citrate lyase
AKT	Protein kinase B
ALDOA	Aldolase A
AML12	Mouse immortalized normal hepatocyte cell line
AMPK	AMP-activated protein kinase
ANRIL	Antisense non-coding RNA in the INK4 locus
ATF4	Activating transcription factor 4
ATGL	Adipose triglyceride lipase
ATP	Adenosine triphosphate
BALB/c	Inbred BALB/c mouse strain
BRL	Buffalo rat liver cell line
C57BL/6J	Inbred C57BL/6J mouse strain
CAFs	Cancer-associated fibroblasts
cAMP	Cyclic adenosine monophosphate
ceRNA	Competing endogenous RNA
ChIP	Chromatin immunoprecipitation
ChIRP	Chromatin isolation by RNA purification
ChREBP	Carbohydrate-responsive element-binding protein
co-IP	Co-immunoprecipitation
CPT1A	Carnitine palmitoyltransferase 1A
CRC	Colorectal cancer
CREB	cAMP-response element binding protein
CRTC2	CREB-regulated transcriptional coactivator 2
db/db	Leptin receptor-deficient diabetic mouse model
DHAP	Dihydroxyacetone phosphate
DIO	Diet-induced obesity (mouse model)
DNL	De novo lipogenesis
DNMT	DNA methyltransferase
DNMT1	DNA methyltransferase 1
DNMT3B	DNA methyltransferase 3 beta
EpCAM	Epithelial cell adhesion molecule
ER	Endoplasmic reticulum
eRNA	Enhancer-associated RNA
EVs	Extracellular vesicles
EZH2	Enhancer of zeste homolog 2
FA	Fatty acid
FASN/FAS	Fatty acid synthase (gene FASN)
FBP	Fructose-bisphosphatase
FFA	Free fatty acid
FLC4	Human hepatoma cell line
FoxO1	Forkhead box protein O1
FXR	Farnesoid X receptor
G3P	Glyceraldehyde 3-phosphate
G6P	Glucose-6-phosphate
G6PC/G6Pase	Glucose-6-phosphatase catalytic subunit (gene G6PC)
GAPDH	Glyceraldehyde-3-phosphate dehydrogenase
GCK	Glucokinase
GDM	Gestational diabetes mellitus
GLOBOCAN	Global Cancer Observatory (IARC/WHO cancer database)
GLP-1R	Glucagon-like peptide 1 receptor
GLUT	Glucose transporter
GLUT1	Glucose transporter 1
GLUT2	Glucose transporter 2
GLUT4	Glucose transporter 4
GPAT	Glycerol-3-phosphate acyltransferase
GPI	Glucose-6-phosphate isomerase
GPR119	G protein-coupled receptor 119
GSK-3β	Glycogen synthase kinase-3β
GYS2	Glycogen synthase 2
H3K27ac	Histone H3 lysine 27 acetylation
H3K27me3	Histone H3 lysine 27 trimethylation
H3K9ac	Histone H3 lysine 9 acetylation
HCC	Hepatocellular carcinoma
HCD	High-cholesterol diet
HDAC	Histone deacetylase
HDAC3	Histone deacetylase 3
HDL	High-density lipoprotein
HDL-C	High-density lipoprotein cholesterol
HepG2	Human hepatocellular carcinoma cell line
HFD	High-fat diet
HFHS	High-fat, high-sucrose diet
HIF-1α	Hypoxia-inducible factor 1α
HK	Hexokinase
HK1	Hexokinase 1
HMGCR	3-Hydroxy-3-methylglutaryl-CoA reductase
HMGCS2	3-Hydroxy-3-methylglutaryl-CoA synthase 2
HNF4α	Hepatocyte nuclear factor 4α
hnRNP	Heterogeneous nuclear ribonucleoprotein
hnRNPA2B1	Heterogeneous nuclear ribonucleoprotein A2/B1
hnRNPL	Heterogeneous nuclear ribonucleoprotein L
HSL	Hormone-sensitive lipase
Huh7	Human hepatocellular carcinoma cell line
IADPSG	International Association of Diabetes and Pregnancy Study Groups
IDF	International Diabetes Federation
IGF-1	Insulin-like growth factor 1
IGF2BP1	Insulin-like growth factor 2 mRNA-binding protein 1
IGF2BP2	Insulin-like growth factor 2 mRNA-binding protein 2
INS-1	Rat pancreatic β-cell line
INSR	Insulin receptor
IR	Insulin resistance
IRS1	Insulin receptor substrate 1
IRS2	Insulin receptor substrate 2
JNK	c-Jun N-terminal kinase
L02	Human normal hepatocyte cell line
LCRE	LXRα-related cis-regulatory element
LDHA	Lactate dehydrogenase A
LDL	Low-density lipoprotein
LDL-C	Low-density lipoprotein cholesterol
LDLR	Low-density lipoprotein receptor
lincRNA	Long intergenic non-coding RNA
lncRNA	Long non-coding RNA
LPL	Lipoprotein lipase
LSD1	Lysine-specific demethylase 1
LXR	Liver X receptor
LXRα	Liver X receptor α
m5C	5-methylcytosine
m6A	N6-methyladenosine
MAFLD	Metabolic (metabolism-associated) fatty liver disease
MASH	Metabolic dysfunction-associated steatohepatitis
MASLD	Metabolic dysfunction-associated steatotic liver disease
MCT	Monocarboxylate transporter
MHCC-97H	Human high-metastatic hepatocellular carcinoma cell line
miRNA	MicroRNA
MPC	Mitochondrial pyruvate carrier
mRNA	Messenger RNA
mTOR	Mechanistic target of rapamycin kinase
mTORC1	Mechanistic target of rapamycin complex 1
MTTP	Microsomal triglyceride transfer protein
NADPH	Nicotinamide adenine dinucleotide phosphate
NAFLD	Nonalcoholic fatty liver disease
NF-κB	Nuclear factor-κB
NFIA	Nuclear factor IA
NLRP3	NOD-like receptor family pyrin domain-containing 3
OAA	Oxaloacetate
ob/ob	Leptin-deficient obese mouse model
Ox-LDL	Oxidized low-density lipoprotein
PBMCs	Peripheral blood mononuclear cells
PC	Pyruvate carboxylase
PDHC	Pyruvate dehydrogenase complex
PDK1	Pyruvate dehydrogenase kinase 1
PEPCK/PCK1	Phosphoenolpyruvate carboxykinase 1 (gene PCK1)
PFK1	Phosphofructokinase 1
PGAM	Phosphoglycerate mutase
PGC-1α	PPARγ coactivator-1α
PGK	Phosphoglycerate kinase
PGK1	Phosphoglycerate kinase 1
PI3K	Phosphatidylinositol 3-kinase
PK	Pyruvate kinase
PKM2	Pyruvate kinase M2
PP2A	Protein phosphatase 2A
PPAR	Peroxisome proliferator-activated receptor
PPARα	Peroxisome proliferator-activated receptor α
PPARγ	Peroxisome proliferator-activated receptor γ
PPP1R3B	Protein phosphatase 1 regulatory subunit 3B
PRC2	Polycomb repressive complex 2
PTBP1	Polypyrimidine tract-binding protein 1
qPCR	Quantitative real-time polymerase chain reaction
RBP	RNA-binding protein
RCT	Reverse cholesterol transport
RHL	Regulator of hyperlipidemia (lncRNA)
RIP	RNA immunoprecipitation
S6K1	Ribosomal protein S6 kinase B1
SAHH	S-adenosylhomocysteine hydrolase
SCD1	Stearoyl-CoA desaturase 1
SHP	Small heterodimer partner
SIRT1	Sirtuin 1
Sirt6	Sirtuin 6
snoRNA	Small nucleolar RNA
SP1	Specificity protein 1
SQLE	Squalene epoxidase
SR-A	Scavenger receptor class A
SR-BI	Scavenger receptor class B type I
SREBP	Sterol regulatory element-binding protein
SREBP-1c	Sterol regulatory element-binding protein 1c
SREBP-2	Sterol regulatory element-binding protein 2
SucCoA	Succinyl-coenzyme A (succinyl-CoA)
T2DM	Type 2 diabetes mellitus
TAG	Triacylglycerol
TC	Total cholesterol
TCA	Tricarboxylic acid (Krebs) cycle
TCF7L2	Transcription factor 7-like 2
TCGA	The Cancer Genome Atlas
TG	Triacylglycerol (triglyceride)
THP-1	Human monocytic leukemia cell line
TLR2	Toll-like receptor 2
VLDL	Very-low-density lipoprotein
WB	Western blot (immunoblot)
WHO	World Health Organization
WNT7A	Wingless-type MMTV integration site family member 7A
XIAP	X-linked inhibitor of apoptosis protein
ZEB1	Zinc finger E-box-binding homeobox 1
α-KG	α-Ketoglutarate
